# Supplementary material for: SpecHLA enables full-resolution HLA typing from sequencing data
Source: Cell Rep Methods. 2023 Sep 14;3(9):100589. doi: 10.1016/j.crmeth.2023.100589 (PMC10545945; doi:10.1016/j.crmeth.2023.100589)
Supplement: Document S1. Figures S1–S4 and Tables S1–S3 [file mmc1.pdf]

Cell Reports Methods, Volume *3*

Supplemental information

SpecHLA enables full-resolution HLA typing from sequencing data

Shuai Wang, Mengyao Wang, Lingxi Chen, Guangze Pan, Yanfei Wang, and Shuai Cheng Li

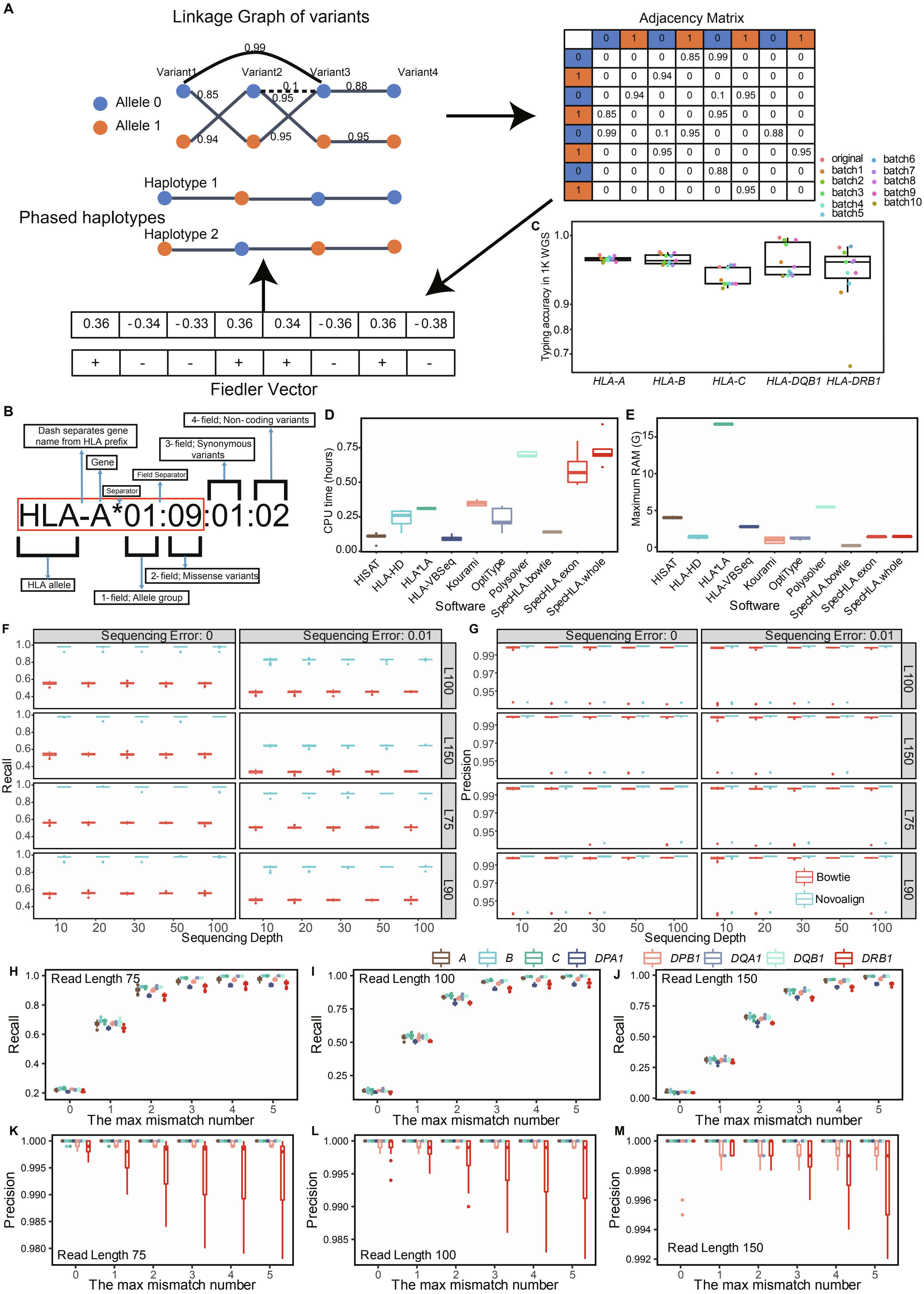

A
Linkage Graph of variants
Variant1
Variant2
Variant3
Variant4
Allele 0
Allele 1
Haplotype 1
Haplotype 2
Phased haplotypes
Adjacency Matrix
0.36 -0.34 -0.33 0.36 0.34 -0.36 0.36 -0.38
+ - - + + - + -
Fiedler Vector
B
Dash separates gene name from HLA prefix
Gene
Field Separator
Separator
3- field; Synonymous variants
4- field; Non- coding variants
HLA-A*01:09:01:02
HLA allele
1- field; Allele group
2- field; Missense variants
C
Typing accuracy in 1K WGS
HLA-A
HLA-B
HLA-C
HLA-DQB1
HLA-DRB1
original
batch1
batch2
batch3
batch4
batch5
batch6
batch7
batch8
batch9
batch10
D
CPU time (hours)
E
Maximum RAM (G)
HISAT
HLA-HD
HLA*LA
HLA-VBSeq
Kourami
OptiType
Polysolver
SpecHLA.bowtie
SpecHLA.exon
SpecHLA.whole
Software
F
G
Sequencing Error: 0
Sequencing Error: 0.01
L100
L150
L75
L90
Recall
Precision
Sequencing Depth
Bowtie
Novoalign
H
I
J
K
L
M
A
B
C
DPA1
DPB1
DQA1
DQB1
DRB1
Read Length 75
Read Length 100
Read Length 150
The max mismatch number

Figure S1: Illustration and evaluation of SpecHLA methodology, related to Figure 1 and STAR Methods.
(A) Illustration of variant phasing with the spectral graph theory. Alleles at each variant are represented as nodes (0 or 1), and the linkage between alleles is depicted as edges. The allele linkage between different variants can be inferred from the sequencing reads, database, genotype frequency, *etc.* The Fiedler vector of the adjacency matrix of the graph is calculated, and the haplotype is inferred by the sign of the Fiedler vector.
(B) Illustration of the naming scheme of HLA alleles. The 1-field indicates the allele group. The 2-field represents the specific HLA protein. The 3-field shows a synonymous DNA substitution within the coding region. The 4-field shows the variants in the non-coding region. Alleles that have identical exon sequences that encode the peptide binding domains will be assigned to the same G group. Full-resolution HLA type indicates the full DNA sequence of the HLA allele.
(C) Comparison of SpecHLA using different reference alleles in 230 WGS 1,000 Genomes samples. The y-axis represents the 2-field HLA typing accuracy, and the scales of 0.9-1.0 and 0.7-0.9 are different. The "*original*" group represents the IMGT representative reference, which concatenates the first recorded allele at each HLA locus. The "*batch**" groups refer to randomly selected alleles.
(D-E) Evaluation of the computational resource consumption of SpecHLA. Different HLA typing methods were run on a simulated sample five times, and the computational resource consumption of each run was measured by CPU time (D) and maximum RAM (E).
(F-G) Comparison of read-binning accuracy using Novoalign and Bowtie2 for alignment in 2,000 simulated samples. The read-binning accuracy was measured by recall (F) and precision (G).
(H-M) Evaluation of the read-binning performance with different maximum mismatch number thresholds (i.e., the hyper-parameter θ). In 200 simulated samples (sequencing depth: 100x; sequencing error rate 1%), we showed the recall with read lengths 75 (H), 100 (I), and 150 (J); and the precision with read lengths 75 (K), 100 (L), and 150 (M).

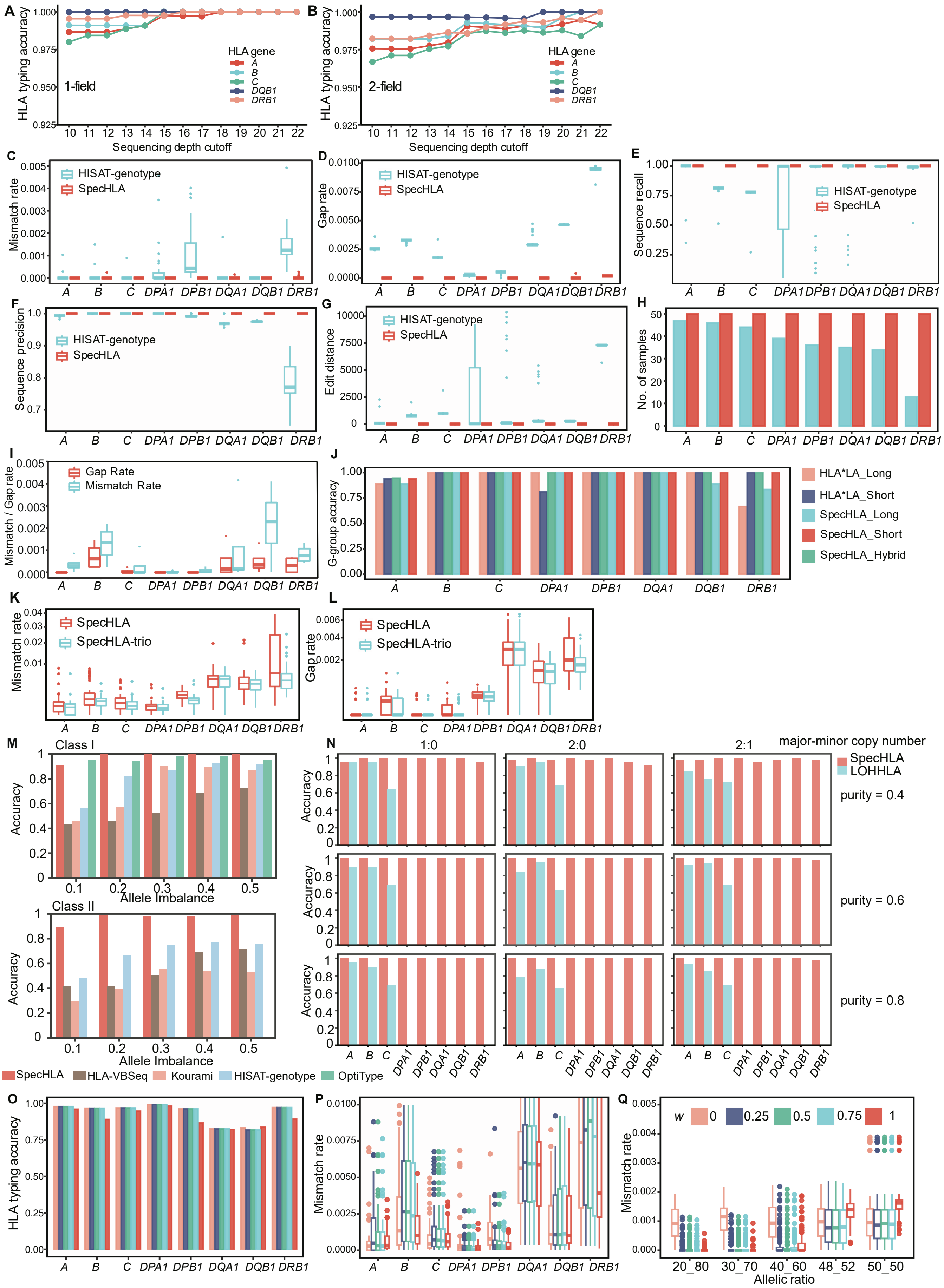

A
B
HLA typing accuracy
1-field
2-field
HLA gene
A
B
C
DQB1
DRB1
Sequencing depth cutoff
C
D
E
F
G
H
HISAT-genotype
SpecHLA
Mismatch rate
Gap rate
Sequence recall
Sequence precision
Edit distance
No. of samples
A
B
C
DPA1
DPB1
DQA1
DQB1
DRB1
I
Gap Rate
Mismatch Rate
Mismatch / Gap rate
J
G-group accuracy
HLA*LA_Long
HLA*LA_Short
SpecHLA_Long
SpecHLA_Short
SpecHLA_Hybrid
K
L
SpecHLA-trio
M
Class I
Class II
Accuracy
Allele Imbalance
N
1:0
2:0
2:1
major-minor copy number
LOHHLA
purity = 0.4
purity = 0.6
purity = 0.8
SpecHLA
HLA-VBSeq
Kourami
HISAT-genotype
OptiType
O
P
Q
w
Allelic ratio
20_80
30_70
40_60
48_52
50_50

Figure S2: Additional evaluation of SpecHLA for HLA typing and LOH inference, related to Figure 2-4 and STAR Methods.
(A-B) Evaluation of SpecHLA using different sequencing depth cutoffs in 230 WGS samples of the 1,000 Genomes Project. The 1-field (A) and 2-field (B) HLA typing accuracy of different genes were displayed separately. Only samples with a sequencing depth higher than the cutoff were included in the evaluation.
(C-H) Comparison of SpecHLA and HISAT-genotype for inferring novel allele sequences in 50 simulated samples. The reconstructed novel alleles were measured by mismatch rate (C), gap rate (D), sequence recall (E), sequence precision (F), and edit distance (G). The number of samples with reconstructed diploid sequences for each gene is shown in (H).
(I) Assessment of trio consistency of SpecHLA in two family trios of the 1,000 Genomes Project. The difference between the inferred sequences of children and parents was measured by mismatch rate and gap rate.
(J) G-group resolution typing accuracy of HLA*LA and SpecHLA using different data protocols in seven HGSVC2 samples and NA12878. HLA*LA was performed on short reads ("*HLA*LA_Short*") and long reads ("*HLA*LA_Long*") separately, while SpecHLA was run with short reads ("*SpecHLA_Short*"), long reads ("*SpecHLA_Long*"), and both short and long reads ("*SpecHLA_Hybrid*").
(K-L) Validation of SpecHLA for incorporating pedigree information using 50 simulated family trios. The performance was measured by mismatch rate (K) and gap rate (L). The y-axis is scaled by the square root. "*SpecHLA-trio*" indicates the incorporation of pedigree information and "*SpecHLA*" means only using reads.
(M) Evaluation of SpecHLA's robustness in allelic imbalance samples using 250 simulated samples. The data had various minor allele ratios (0.1, 0.2, 0.3, 0.4, and 0.5), with 50 replicates for each ratio. The 2-field HLA typing accuracy of MHC class I and II genes was shown separately.
(N) Evaluation of the LOH detection of SpecHLA with different tumor purity and ploidy using simulated data. The LOH detection accuracy of LOHHLA and SpecHLA was compared in a total of 450 samples from nine PE datasets with different tumor purity (0.4, 0.6, 0.8) and major-minor copy numbers (1:0, 2:0, 2:1), with 50 replicates for each dataset (sequencing depth: 100x; read length: 150bp; sequencing error rate: 0%).
(O-Q) Evaluation of SpecHLA with different values of the hyper-parameter *w*. The hyper-parameter *w* adjusts the weight of genotype frequency information. (O) 2-field HLA typing accuracy in 200 LOH samples generated by cancer cell lines and normal cell data. (P) Mismatch rate in 80 allelic imbalance simulated samples. In each sample, the depths of the two haplotypes were 20x and 80x. (Q) Mismatch rate of SpecHLA in 100 simulated novel-allele samples with different allelic depths. E.g., "*30_70*" shows the depths of the two haplotypes were 30x and 70x.

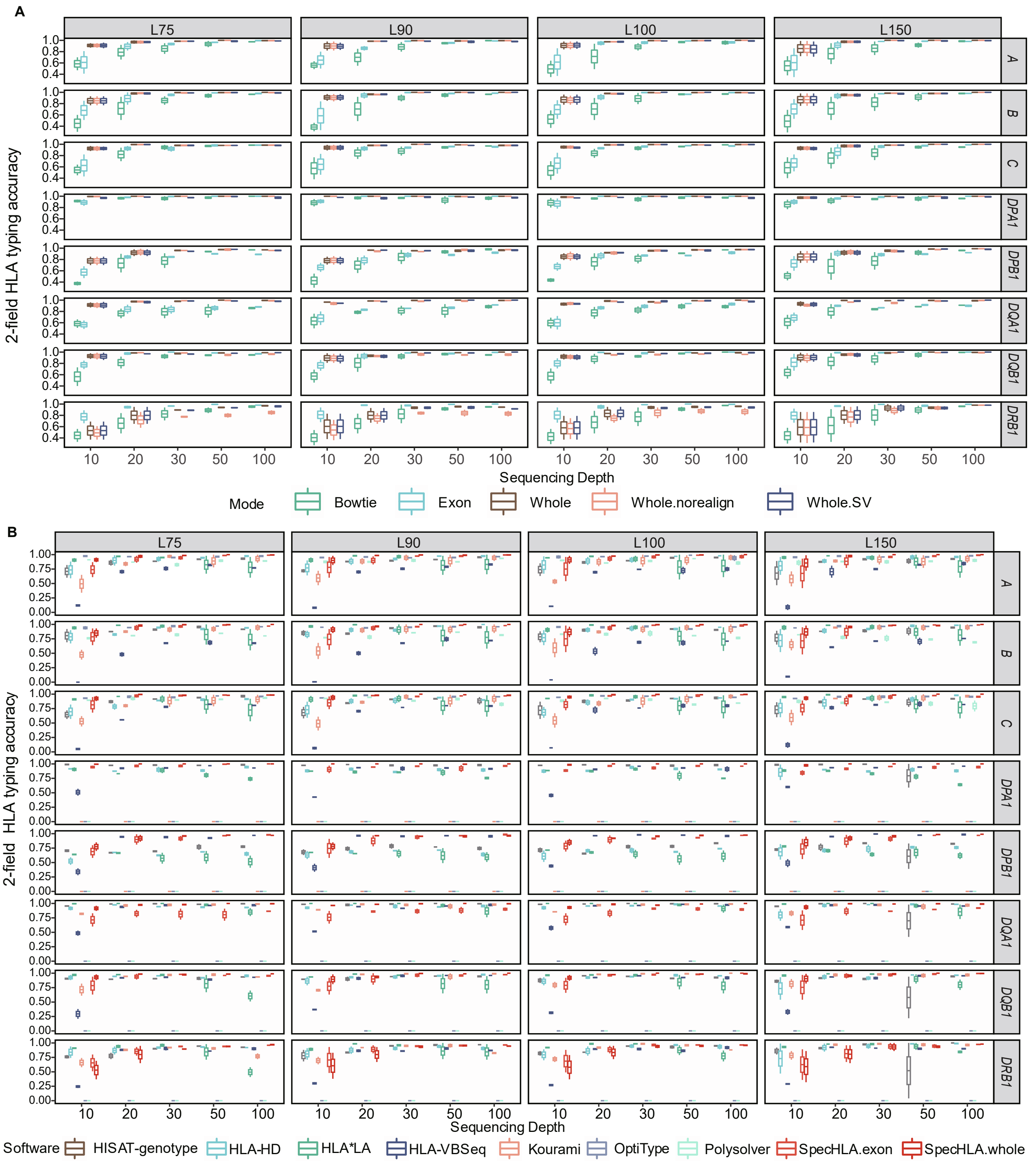

Figure S3: Evaluation of SpecHLA for 2-field typing using the 2,000 simulated samples, related to Figure 2. **(A)** Comparison of SpecHLA with different modes for 2-field typing. The 2-field typing accuracy of SpecHLA in different modes with varying sequencing depths and read lengths was displayed separately. "*Bowtie*" indicates the usage of Bowtie2 for alignment in the read binning step. "*Exon*" and "*whole*" represent focusing on the exon region and the full-length allele, respectively. "*Whole.norealign*" denotes discarding the local assembly step, while "*Whole.SV*" involves considering long InDels.

(B) Comparison of SpecHLA and other methods for 2-field typing. The 2-field typing accuracy of different software with different sequencing depths and read lengths was displayed separately. "*SpecHLA.exon*" refers to reconstructing exons and obtaining typing annotation based on exons, while "*SpecHLA.whole*" focuses on the full-length HLA alleles.

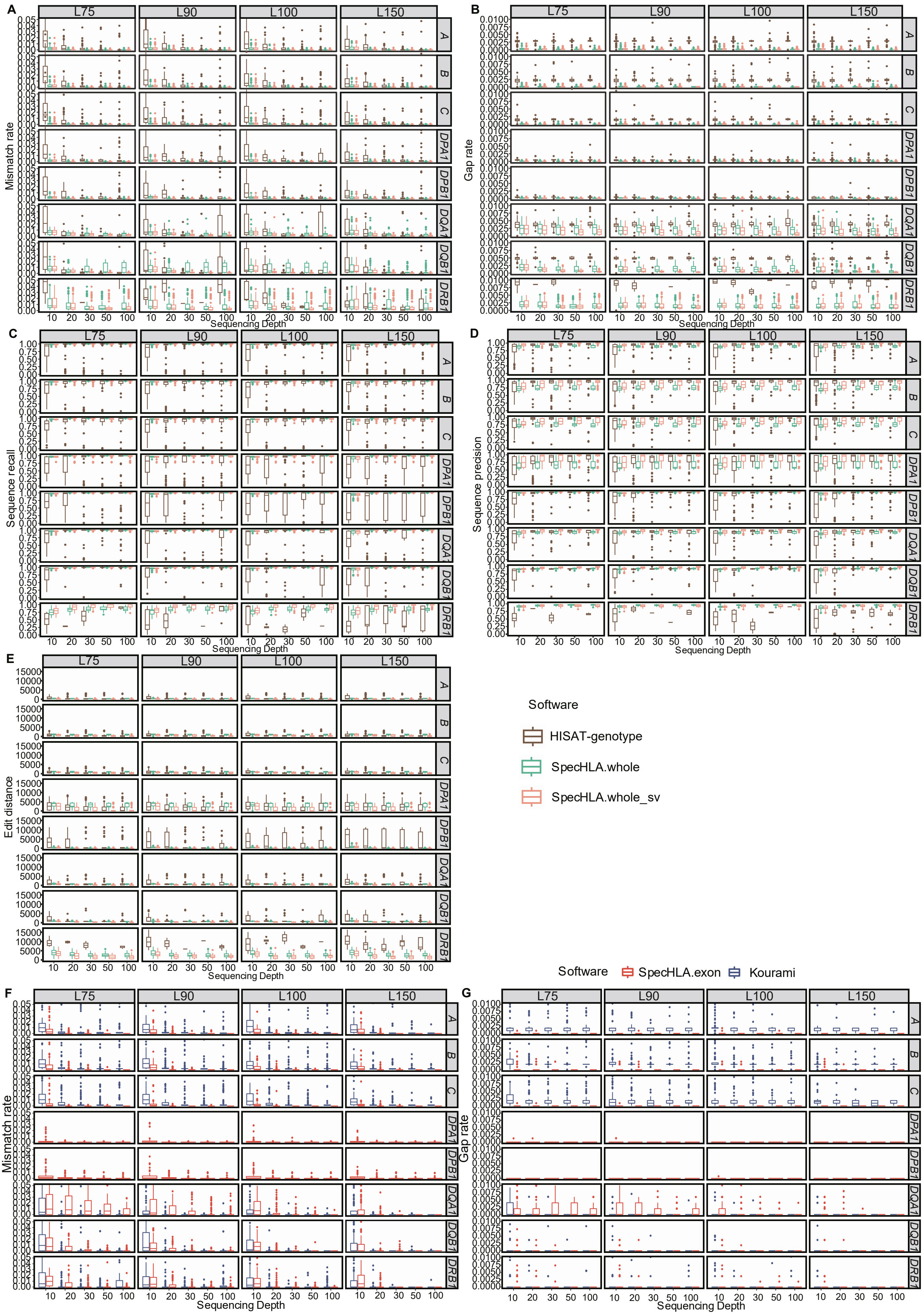

A
B
C
D
E
F
G
L75
L90
L100
L150
A
B
C
DPA1
DPB1
DQA1
DQB1
DRB1
Mismatch rate
Gap rate
Sequence recall
Sequence precision
Edit distance
Sequencing Depth
10
20
30
50
100
Software
HISAT-genotype
SpecHLA.whole
SpecHLA.whole_sv
SpecHLA.exon
Kourami

Figure S4: Evaluation of SpecHLA for sequence reconstruction using the 2,000 simulated samples, related to Figure 2.
(A-E) Comparison of SpecHLA and HISAT-genotype for full-resolution HLA typing. The performance was measured by mismatch rate (A), gap rate (B), sequence recall (C), sequence precision (D), and edit distance (E). The accuracy was calculated for different read lengths and sequencing depths separately. "*SpecHLA.whole*" denotes performing HLA typing on full-length allele while ignoring long InDels, and "*SpecHLA.whole_sv*" involves identifying and phasing long InDels.
(F-G) Comparison of SpecHLA (exon mode) and Kourami for exonic sequence inference. The exonic sequence was assessed by mismatch rate (F) and gap rate (G). The accuracy was calculated for different read lengths and sequencing depths separately. Only exons 2 and 3 for MHC class I genes and exon 2 for MHC class II genes were considered.

Gene	No. of alleles (exon)	No. of alleles (full-length)	Representative Allele	Highly divergent regions
HLA-A	131	1141	A*01:01:01:01	
HLA-B	230	1512	B*07:02:01:01	1500-1800
HLA-C	80	1323	C*01:02:01:01	
HLA-DMA	2	5		
HLA-DMB	2	8		
HLA-DOA	1	7		
HLA-DOB	3	10		
HLA-DPA1	4	86	DPA1*01:03:01:01	
HLA-DPA2	2	5		
HLA-DPB1	55	441	DPB1*01:01:01:01	2150-2800, 9724-9950, 10000-10500
HLA-DPB2	2	5		
HLA-DQA1	16	140	DQA1*01:01:01:01	2900-3202, 3345-3812, 3900-4150, 4999-5320, 5800-6150, 6550-6800
HLA-DQA2	7	15		
HLA-DQB1	24	239	DQB1*02:01:01	2450-2650, 3205-4115, 4100-4250, 4400-4600, 4750-5200, 5300-5700, 5800-6100
HLA-DRA	1	28		
HLA-DRB1	816	816	DRB1*01:02:01:01	2250-2450, 2774-3266, 3292-5120, 6700-7100, 7192-7540, 8893-9299, 10032-10305, 11020-11728
HLA-DRB3	77	77		
HLA-DRB4	24	24		
HLA-DRB5	26	26		
HLA-E	10	41		
HLA-F	6	44		
HLA-G	6	40		
HLA-H	6	9		
HLA-HFE	1	3		
HLA-J	2	9		
HLA-K	3	6		
HLA-L	2	5		
HLA-MICA	13	15		
HLA-MICB	12	31		
HLA-N	1	5		
HLA-P	3	5		
HLA-S	4	7		
HLA-T	3	8		
HLA-TAP1	2	6		
HLA-TAP2	1	8		
HLA-U	4	5		
HLA-V	1	3		
HLA-W	5	11		
HLA-Y	3	3		

Table S1: HLA database profile used for the reads binning and reads alignment procedures of SpecHLA, related to STAR Methods.

The first column presents the 39 HLA genes included in the HLA database. The second and third columns display the numbers of alleles in the HLA database for reads binning of exon typing and full-length typing, respectively. After downloading the IMGT/HLA database, alleles with a population frequency equal to zero were removed for the eight focused genes, while all alleles were retained for other genes. Additionally, alleles of *HLA-DRB2, -DRB6, -DRB7, -DRB8,* and *-DRB9* were discarded. For exon typing, the database retains only one allele from the alleles with the same 2-field designation for each HLA gene, except for *HLA-DRB1*. The fourth column indicates the IMGT representative alleles used in the read-binning process. The assigned reads are separately projected to the representative allele of each gene. The last column shows the interval of highly divergent regions of the IMGT representative alleles. SpecHLA assembles the reads mapped to such regions to enhance the quality of read alignment.

Data Protocol	Software	Command
PE	DWGSIM v0.1.13	dwgsim -e 0 -E 0 -1 150 -2 150 -C 10 -r 0
10x	LRSIM v1.0	perl simulateLinkedReads.pl -r -p -x 1 -f 3 -t 1 -m 10 -o -c LRSIM/test/fragmentSizesList
Hi-C	sim3C v0.2	sim3C –dist uniform -n 37574 -l 150 -e NlaIII -m hic
PacBio	PBSIM v1.0.3	pbsim –data-type CLR –seed 88 –accuracy-mean 0.85 –accuracy-min 0.80 –depth 10 –model_qc model_qc_clr
ONT	NanoSim v3.1.0	simulator.py genome -c human_NA12878_DNA_FAB49712_guppy/training -rg -o -max 5000 -n 4000 –seed 66

Table S2: Parameters for simulating different data protocols, related to STAR Methods. We simulated data using these protocols for each one of the 50 simulated individuals.

Software	Version	Data	Parameter
SpecHLA	v1.0.1	WGS	-u 0 -p $pop -m 5
		WES	-u 1 -p $pop -k 4
		RNA-seq	-u 1 -p $pop
		HGSVC2 Full-length	-u 0 -p nonuse
		HGSVC2 hybrid	-u 0 -b 0 -1 $fq1 -2 $fq2 -t $pacbio
		trios for trio-consistency assessment	-u 0 -m 4
		trios for pedigree info integration	-u 0 -m 4 -b 0
		Simulated data	-u 0 -q 1 -s 3 -r 0.1 -p nonuse
		Simulated data using rare alleles	-u 0 -p nonuse -m 10
		long-read only	long_read_typing.py -r $long_reads -n $sample
		IHWG cell line	-u 1 -s 3 -m 5 -k 3 -z True
		LOH	-u 1 -r 0.05 -w 0.1 -p Unknown
HISAT-genotype	v1.3.2	Simulated data/HGSVC2	–base hla –locus-list A,B,C,DPA1,DPB1,DQA1,DQB1,DRB1 –assembly -1 $fq1 -2 $fq2 –read-len $readlen
		WGS/WES/RNA-seq	–base hla –locus-list A,B,C,DPA1,DPB1,DQA1,DQB1,DRB1 -1 $fq1 -2 $fq2 –read-len $readlen
HLA-HD	v1.4.0	WGS/WES/RNA-seq/Simulated data	-m $readlen -c 0.95
HLA-LA	v1.0.2	WGS/WES/Simulated data	default
		long-read	–longReads pacbio
HLA-VBseq	v2	WGS/WES/Simulated data	–alpha_zero 0.01 –is_paired -d 4
Kourami	v0.9.6	WGS/WES/simualtion	default
OptiType	v1.3.1	WGS/WES/Simulated data	default
		RNA-seq	–rna
Polysolver	v1.0.0	WES/Simulated data	default
arcasHLA	v0.5.0	RNA-seq	arcasHLA genotype $fq1 $fq2 -g A,B,C,DPA1,DPB1,DQB1,DQA1,DRB1
LOHHLA	v1.1.6	LOH	default
DASH	training.xgboost_model. 2021_05_10.p	LOH	default

Table S3: Parameter settings of benchmark tools for HLA typing and LOH detection, related to STAR Methods.
For all benchmark tools, unless mentioned otherwise, any unmentioned parameters were set to their default values.
