# Supplementary material for: SpecHLA enables full-resolution HLA typing from sequencing data
Source: Cell Rep Methods. 2023 Sep 14;3(9):100589. doi: 10.1016/j.crmeth.2023.100589 (PMC10545945; doi:10.1016/j.crmeth.2023.100589)
Supplement: Document S2. Article plus supplemental information [file mmc2.pdf]

Cell Reports
Methods

Report

SpecHLA enables full-resolution HLA typing from sequencing data

Graphical abstract

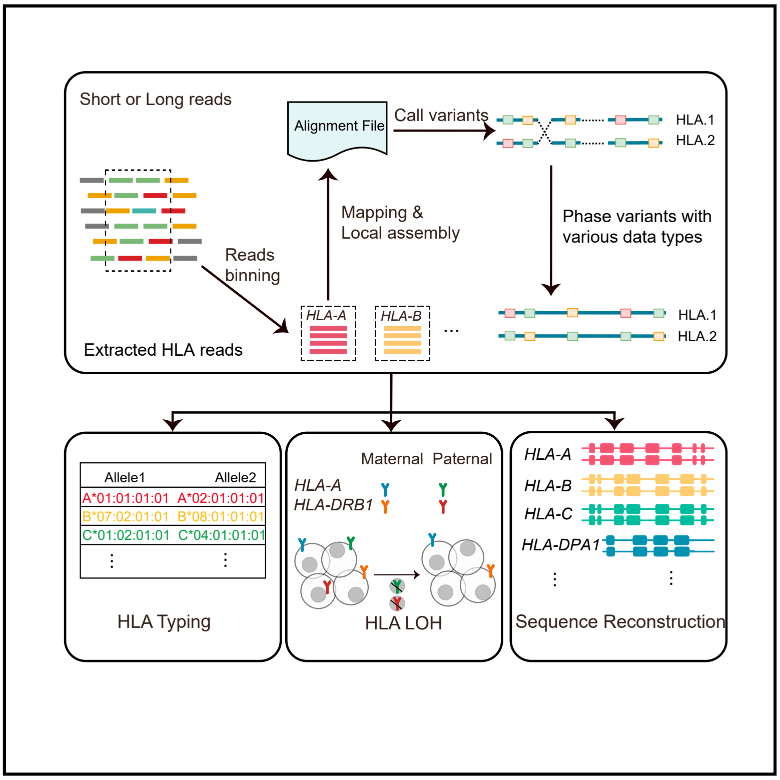

Highlights

- SpecHLA accurately reconstructs diploid sequences of HLA genes
- SpecHLA accepts diverse sequencing data protocols
- SpecHLA can leverage pedigree data and genotype frequency to refine typing
- SpecHLA accurately detects HLA loss-of-heterozygosity events

Authors

Shuai Wang, Mengyao Wang, Lingxi Chen, Guangze Pan, Yanfei Wang, Shuai Cheng Li

Correspondence

shuaicli@cityu.edu.hk

In brief

Wang et al. present SpecHLA, a software package leveraging reads binning and local assembly to achieve accurate full-resolution HLA typing and loss-of-heterozygosity detection. SpecHLA accepts various sequencing data protocols and can incorporate pedigree data and genotype frequency for better performance.

Wang et al., 2023, Cell Reports Methods *3*, 100589
September 25, 2023 © 2023 The Author(s).
https://doi.org/10.1016/j.crmeth.2023.100589

CellPress

Cell Reports Methods

CellPress
OPEN ACCESS

Report

SpecHLA enables full-resolution HLA typing from sequencing data

Shuai Wang,[1,2] Mengyao Wang,[1,2] Lingxi Chen,[1] Guangze Pan,[1] Yanfei Wang,[1] and Shuai Cheng Li[1,3,*]
[1]City University of Hong Kong, Department of Computer Science, Kowloon, Hong Kong
[2]These authors contributed equally
[3]Lead contact
*Correspondence: shuaicli@cityu.edu.hk
https://doi.org/10.1016/j.crmeth.2023.100589

MOTIVATION HLA genes play a crucial role in the regulation of the adaptive immune system, and HLA typing is an essential approach for understanding numerous diseases. Nevertheless, existing HLA typing methods are underdeveloped due to two primary challenges. First, the high similarity among alleles of different HLA genes poses a challenge. Second, the exceptional polymorphism of HLA alleles within the same gene further complicates the typing process. In this study, we utilize fine-tuned reads binning to overcome the issue of allele similarity and leverage local assembly to address the challenge of allele polymorphism, allowing accurate read alignment. By identifying and phasing variants based on enhanced read alignment, we can accurately reconstruct the diploid sequences of HLA genes. Additionally, the genotype frequency of phased variants allows us to detect HLA loss of heterozygosity.

SUMMARY

Reconstructing diploid sequences of human leukocyte antigen (HLA) genes, i.e., full-resolution HLA typing, from sequencing data is challenging. The high homogeneity across HLA genes and the high heterogeneity within HLA alleles complicate the identification of genomic source loci for sequencing reads. Here, we present SpecHLA, which utilizes fine-tuned reads binning and local assembly to achieve accurate full-resolution HLA typing. SpecHLA accepts sequencing data from paired-end, 10×-linked-reads, high-throughput chromosome conformation capture (Hi-C), Pacific Biosciences (PacBio), and Oxford Nanopore Technology (ONT). It can also incorporate pedigree data and genotype frequency to refine typing. In 32 Human Genome Structural Variation Consortium, Phase 2 (HGSVC2) samples, SpecHLA achieved 98.6% accuracy for G-group-resolution HLA typing, inferring entire HLA alleles with an average of three mismatches fewer, ten gaps fewer, and 590 bp less edit distance than HISAT-genotype per allele. Additionally, SpecHLA exhibited a 2-field typing accuracy of 98.6% in 875 real samples. Finally, SpecHLA detected HLA loss of heterozygosity with 99.7% specificity and 96.8% sensitivity in simulated samples of cancer cell lines.

INTRODUCTION

Human leukocyte antigen (HLA) genes encode the major histocompatibility complex (MHC) molecules essential in adaptive immune system regulation.[1] HLA typing assists in organ transplantation and preimplantation genetic diagnosis[2] and yields insights into the molecular mechanism of autoimmune disorders,[3] infectious diseases,[4] cancer immunotherapy,[5] etc. Many HLA typing methods identify the most compatible pair of alleles for each gene that match the sequencing data from the database.[6] For instance, OptiType[7] maximizes the number of mapped reads on the inferred alleles by employing integer linear programming. PolySolver[8] selects alleles via a Bayesian model by integrating base qualities of aligned reads, observed insert sizes, and ethnicity-dependent allele frequency. HLA-VBSeq[9] optimizes both read alignments to alleles and relative quantities of reads based on variational Bayesian inference. HLA-HD[10] maps reads to exons and introns of alleles in the database and utilizes weighted read counts to choose suitable allele pairs. HLA*PRG[11] transforms the allele database to a population reference graph and applies the read-to-graph alignment algorithm to infer the most likely allele pair. The method arcasHLA[12] aligns reads to the de Bruijn graph constructed from the reference transcriptome and then identifies the allele pair using the *k*-mer structure. HLA*LA[13] projects the linear read alignment on the reference graphs, allowing allele inference from short- or long-read data. However, these methods have several limitations: (1) they are imprecise if the target alleles are incomplete or absent from the

This is an open access article under the CC BY license (http://creativecommons.org/licenses/by/4.0/).

database[14]; (2) they are inefficient in adopting the ever-expanding list of alleles, as the HLA database updates frequently[6]; (3) they are inaccurate for the high level of resolution[14]; and (4) they cannot infer the exact sequence of HLA alleles. Recently, researchers have attempted to reconstruct the diploid sequences of HLA genes, i.e., full-resolution HLA typing. Lee and Kingsford developed Kourami[6] to assemble HLA exons using the modified partial-order graph. Kim et al. implemented HISAT-genotype[15] that splits aligned reads into *k*-mers and resolves assembly ambiguities to generate full-length HLA alleles.

Assembly and alignment are indispensable for full-resolution HLA typing. The assembly-based approach is to assemble reads into haplotype-resolved sequences, and the alignment-based approach is to align reads to a reference allele and then identify and phase the variants. Nevertheless, assembly and alignment both suffer from two challenges. First, the alleles of different HLA genes possess a high similarity,[7] making it complicated to identify the genomic sources of reads unambiguously. Hence, typing an HLA locus would be interfered with by reads derived from other homologous loci. For instance, reads originating from the *HLA-A*-like pseudogene *HLA-Y* hamper typing *HLA-A*.[10] Although a previous method attempted to discard the reads potentially generated from other loci to type the target locus, the method cannot reconstruct HLA allele sequences and is not open source.[16] Second, HLA alleles of the same gene are exceptionally polymorphic; consequently, the reads might be highly different from the reference allele. Due to the limitation of scoring systems of alignment algorithms, homologous reads are possibly mapped to alternative coordinates. Moreover, the highly variable reads render assembly highly fragmented and prone to error.[17]

Here, we present a software package, SpecHLA, for accurate full-resolution HLA typing on *HLA-A*, *-B*, *-C*, *-DPA1*, *-DPB1*, *-DQA1*, *-DQB1*, and *-DRB1* genes. The package adopts fine-tuned reads binning and local assembly for precise read alignment and reconstructs complete diploid sequences of HLA loci through variant phasing. SpecHLA determines the reads belonging to an HLA locus by aligning the reads to known HLA alleles to solve the first challenge. Furthermore, SpecHLA assembles reads mapped to each highly divergent region into contigs independently and realigns the reads to the reference allele through the assembled contigs to overcome the second challenge. SpecHLA achieved 97.6%, 98.4%, and 99.1% 2-field typing accuracies in 230 whole-genome sequencing (WGS) samples, 183 whole-exome sequencing (WES) samples, and 462 RNA sequencing (RNA-seq) samples from the 1000 Genomes Project, respectively. In the 32 WGS samples from the Human Genome Structural Variation Consortium, Phase 2 (HGSVC2) Project,[18] SpecHLA achieved 98.6% HLA typing accuracy at G group resolution. Also, on average, it reconstructed entire HLA alleles with three mismatches fewer, ten gaps fewer, and 590 bp less edit distance than HISAT-genotype compared with ground-truth per allele.

Furthermore, the development of sequencing technologies makes it necessary to support HLA typing for multiple data protocols. Unlike most methods that are limited to a single data protocol, SpecHLA accepts the sequencing data of paired-end (PE), 10× Genomics,[19] high-throughput chromosome conformation capture (Hi-C),[20] Pacific Biosciences (PacBio), and Oxford Nanopore Technology (ONT).[21] Additionally, it has been recognized that utilizing pedigree relations[22] and genotype frequency[23] can guide variant phasing and help to produce more precise diploid sequences. SpecHLA can incorporate the pedigree relations and combine genotype frequency to generate more reliable HLA typing results.

HLA loss-of-heterozygosity (LOH) events frequently occur in patients with cancer and can cause immune evasion during cancer evolution.[24] For example, loss of *HLA-C*∗08:02 was proposed to result in immune evasion in metastatic colorectal cancer.[25] Computational methods have been developed to identify LOH events from sequencing data. LOHHLA[26] infers LOH by counting the uniquely mapped reads onto the typed alleles. DASH[27] applies machine learning to predict LOH using features such as adjusted b-allele frequency, sequencing depth ratio, consistency of sequencing depth, etc. SpecHLA detects LOH events based on the genotype frequency of phased variants. SpecHLA exhibited 99.7% specificity and 96.8% sensitivity for LOH detection in 300 simulated samples from cancer cell lines with various tumor purities. SpecHLA enables accurate full-resolution HLA typing and LOH detection in MHC class I and II genes from different data types.

RESULTS

Overview of SpecHLA

SpecHLA collects the sequencing reads of the HLA region (Figure 1A) and then maps the collected reads to the HLA allele database and bins the reads to HLA loci according to their sequence identity with the mapped alleles (Figure 1B). At each HLA locus, the first recorded allele in the IMGT/HLA database is utilized as the IMGT representative reference. We align the binned reads to the IMGT representative reference. Then, we extract the reads aligning to each highly divergent region of the reference and assemble them into contigs independently (Figure 1C). The extracted reads are realigned to the IMGT representative reference coordinated by the assembled contigs. We next call small variants and long insertions or deletions (indels) (Figure 1D) and phase the variants across the gene. Small variants are phased using spectral graph theory[28] (Figures 1E and S1A), and long indel variants are distinguished with supporting reads. We generate diploid sequences of each HLA locus through the phased variants. The generated sequence is aligned to the IMGT/HLA database[29] for the official designation, considering the ethnicity-dependent allele frequency (see Figure S1B for the naming scheme of HLA alleles). To detect LOH events, we infer the sequence frequency at each HLA locus based on the genotype frequency of phased variants (Figure 1F). We validated SpecHLA for 2-field typing, sequence reconstruction, and LOH detection using both actual and simulated samples (Figure 1G). The functionalities of SpecHLA and the state-of-the-art tools related to HLA are summarized in Figure 1H.

Additionally, we evaluated the methodology of SpecHLA in various aspects. First, we investigated the impact of different representative reference alleles on SpecHLA's performance. We conducted separate runs of SpecHLA in the 230 WGS samples from the 1000 Genomes Project, using ten randomly chosen

CellPress
OPEN ACCESS

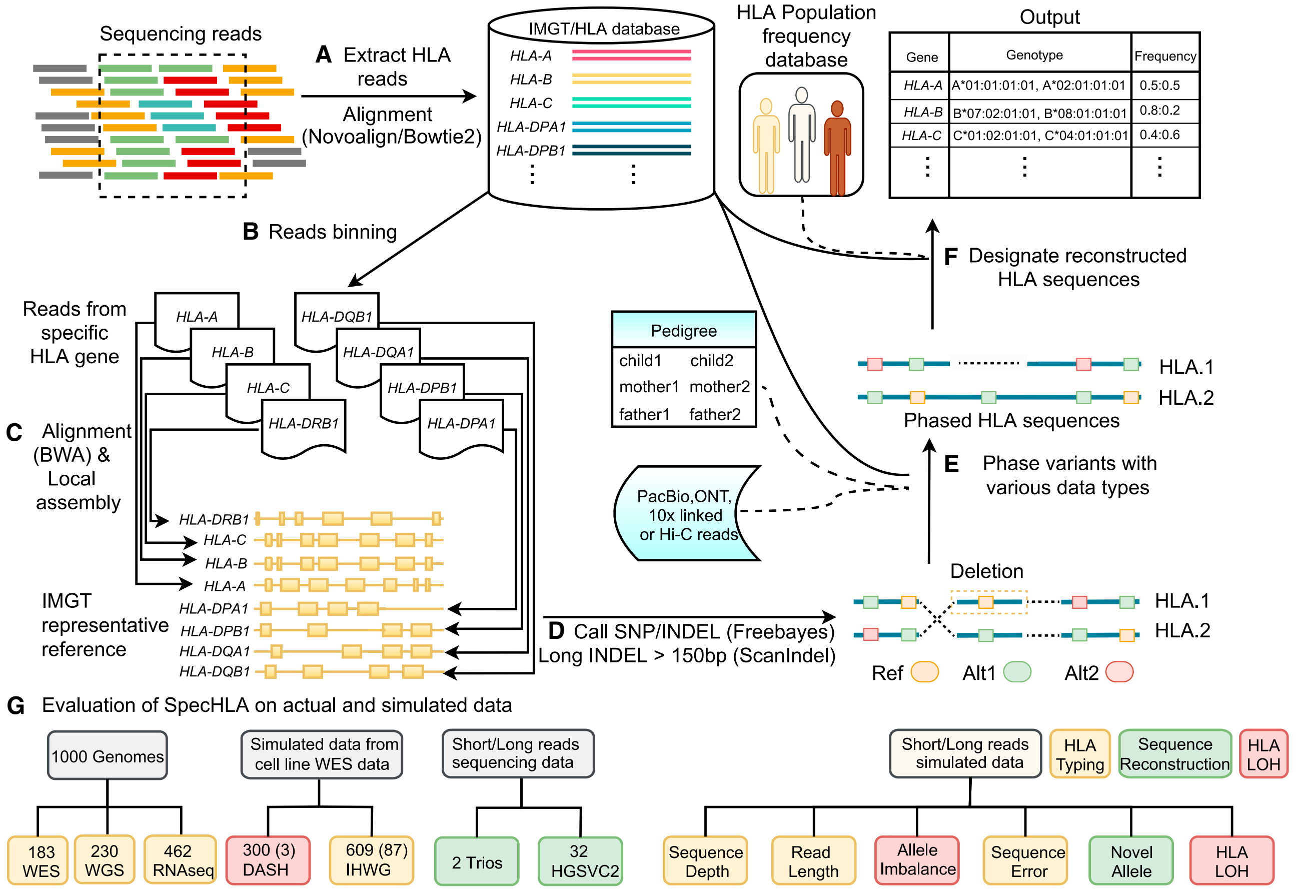

H Functionalities of state-of-the-art tools in HLA research

Method	Sequence Reconstruction		HLA Gene		Sequence Strategy						Complementary Read Info			Additional Info		
	Exon	Entire gene	Class I	Class II	WGS	WES	RNA-seq	PE	SE	Long-read	10x	Hi-C	Long-read	Pedigree	Imbalance	LOH
SpecHLA	√	√	√	√	√	√	√	√		√	√	√	√	√	√	√
OptiType			√		√	√	√	√	√							
Polysolver			√			√		√								
HLA-VBseq			√	√	√	√		√	√							
Kourami	√		√	√	√	√		√								
HISAT-genotype	√	√	√	√	√	√	√	√	√							
HLA*LA			√	√	√	√		√		√						
HLA-HD			√	√	√	√	√	√								
arcasHLA			√	√			√	√	√							
LOHHLA			√		√	√		√								√
DASH			√		√	√		√								√

Figure 1. The SpecHLA workflow

(A) Extract the reads mapped to the HLA region and align them to the HLA allele database.
(B) Bin reads to the HLA loci according to their sequence identity with known alleles.
(C) Align the binned reads to the IMGT representative reference and perform local realignment.
(D) Call SNVs and short indels using Freebayes and call long indels using ScanIndel.
(E) Reconstruct the diploid sequences by phasing the variants.
(F) Compare the reconstructed sequences with the database to report the official designations and compute sequence frequencies to detect LOH events.
(G) Evaluation setting of SpecHLA on actual and simulated data.
(H) Functionalities of state-of-the-art tools in HLA research. A checkmark (√) indicates that the software has the feature. PE refers to paired-end reads, while SE indicates single-end short reads. The term "pedigree" indicates the incorporation of pedigree information, while "imbalance" represents the adoption of genotype frequency in samples with allelic imbalance.
See also Figure S1.

reference alleles and the default IMGT representative reference. For most genes, the 2-field HLA typing results showed no significant differences when using different reference alleles, except for the *HLA-DRB1* gene (see Figure S1C). Notably, when using the randomly selected allele DRB1*09:01:02:02 as the reference, the accuracy of *HLA-DRB1* significantly decreased to 65.3%. Conversely, employing the default reference allele resulted in relatively high accuracy across all genes, suggesting that

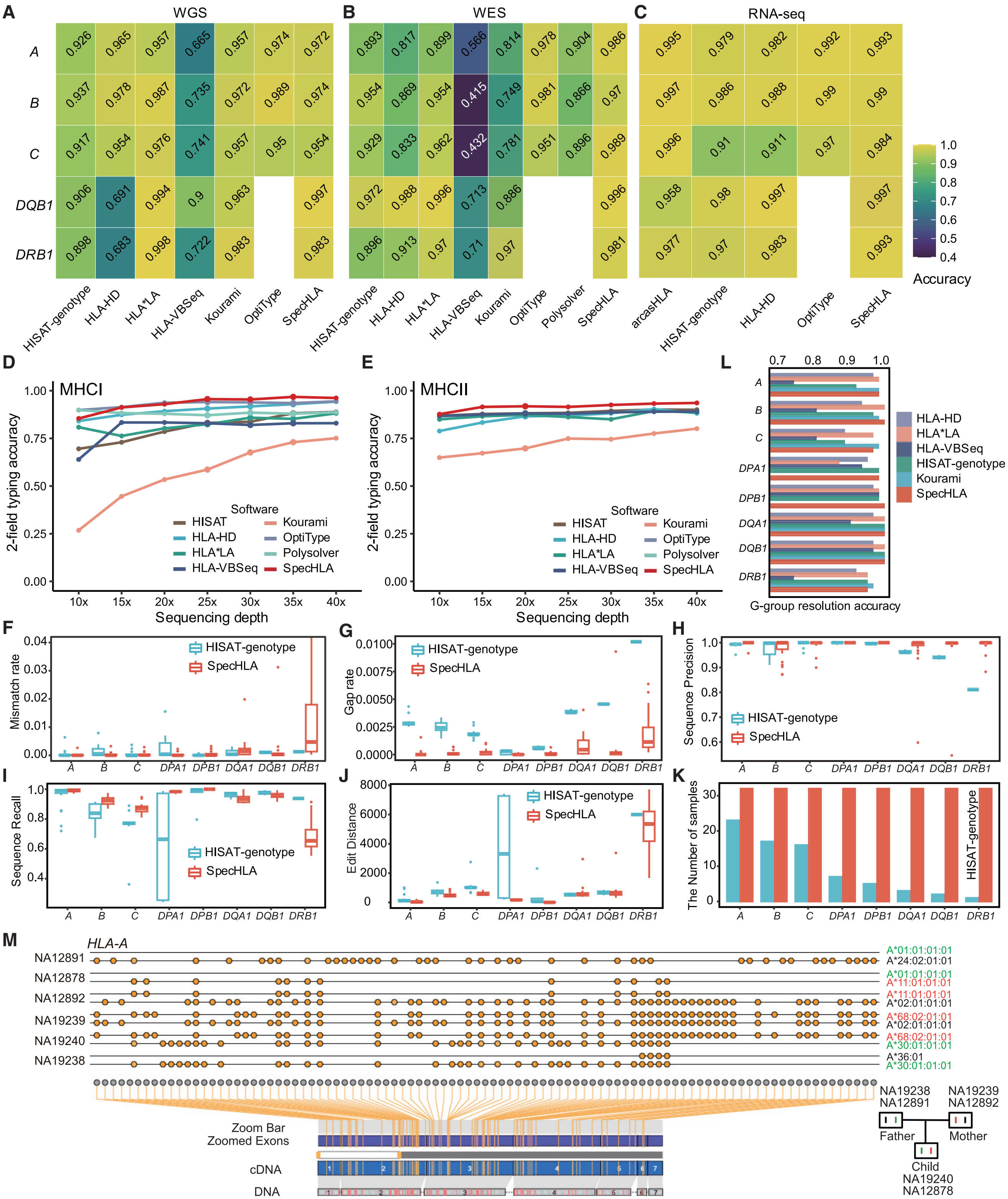

(legend on next page)

SpecHLA exhibits greater robustness when utilizing the default reference allele. Second, in terms of computational resource consumption, SpecHLA exhibited relatively higher CPU time requirements but demanded less memory compared with other tools (Figures S1D and S1E). Third, we evaluated the effect of alignment tools on the read-binning performance in 2,000 simulated samples. When using Novoalign, the read-binning step exhibited an average precision of 99.8% and a recall of 89.4%. Conversely, employing Bowtie2 resulted in an average precision of 99.6% and a recall of 49.9% (Figures S1F and S1G). The result demonstrated that Novoalign outperforms Bowtie2 in terms of read-binning accuracy.

SpecHLA is accurate in 2-field HLA typing

First, SpecHLA performed accurately in 2-field HLA typing on actual data. We evaluated SpecHLA with 230 WGS, 183 WES, and 462 RNA-seq samples containing validated HLA typing results for the five genes, *HLA-A*, *-B*, *-C*, *-DQB1*, and *-DRB1* from the 1000 Genomes Project (Figures 2A–2C). SpecHLA achieved an average typing accuracy of 98.6% in the 875 samples, while HISAT-genotype, HLA-HD, HLA*LA, HLA-VBSeq, Kourami, OptiType, Polysolver, and arcasHLA exhibited accuracies of 94.7% (875 samples); 93.1% (875 samples); 97.1% (413 samples); 66.3% (413 samples); 90.7% (413 samples); 97.8% (875 samples); 88.9% (183 samples); and 98.5% (462 samples), respectively. SpecHLA demonstrated the highest average accuracy across a range of experiments involving different genes and sequencing protocols on actual data.

Second, the typing accuracy of SpecHLA was robust with varying sequencing depths. The 230 WGS samples have sequencing depths ranging from 10× to 49× in the MHC region. We observed that the 2-field typing accuracy of SpecHLA raised from 98.1% to 99.7%, with the depth cutoff increasing from 10× to 22× (Figures S2A and S2B). Moreover, we subsampled the 87 IHWG cell line WES samples[30] for various sequencing depths (10×, 15×, 20×, 30×, 35×, and 40×). The average MHC class I gene typing accuracies of SpecHLA, HISAT-genotype, HLA-HD, HLA*LA, HLA-VBSeq, Kourami, OptiType, and Polysolver across different depths were 93.4%, 80.7%, 90%, 82.7%, 80.2%, 57%, 93%, and 88.3%, respectively (Figures 2D and 2E). The average MHC class II gene typing accuracies of SpecHLA, HISAT-genotype, HLA-HD, HLA*LA, HLA-VBSeq, and Kourami were 91.7%, 88.7%, 86.1%, 86.9%, 88.1%, and 72.7%, respectively. SpecHLA had a typing accuracy that increased steadily with the increase of sequencing depths.

Third, simulated data demonstrated that SpecHLA performed reliably on data of various depths, read lengths, and sequencing error rates. We simulated 40 PE datasets with different depths (10×, 20×, 30×, 50×, and 100×), read lengths (75, 90, 100, and 150 bp), and sequencing error rates (0% and 1%). 50 replicates were generated for each dataset, resulting in 2,000 samples in total. Using these datasets, we first evaluated SpecHLA with different parameter settings (Figure S3A). The mean 2-field typing accuracies of SpecHLA "Bowtie," "Exon," "Whole.norealign," "Whole," and "Whole.SV" modes were 82%, 90.2%, 94.2%, 94.8%, and 94.8%, respectively. The result showed that (1) the local assembly was effective; (2) Novoalign worked better than Bowtie2 for reads binning; (3) full-length typing was more accurate than exon typing; and (4) the 2-field typing performance was similar with or without considering long indels. Hereafter, we benchmarked SpecHLA under the "Whole" mode with other methods. SpecHLA outperformed other methods for MHC class I and II genes in the 2,000 samples (Figure S3B). SpecHLA, HISAT-genotype, HLA-HD, HLA*LA, HLA-VBSeq, Kourami, OptiType, and Polysolver had average typing accuracies on MHC class I genes of 97.2%, 87.3%, 89%, 89%, 59.8%, 84%, 95.7%, and 85.5%, respectively. Also, SpecHLA, HISAT-genotype, HLA-HD, HLA*LA, HLA-VBSeq, and Kourami exhibited average typing accuracies on class II genes of 94.4%, 89.3%, 87%, 84.7%, 83.2%, and 92%, respectively.

SpecHLA accurately reconstructs HLA sequences

First, SpecHLA reconstructed entire HLA alleles of higher quality than HISAT-genotype on actual and simulated data. We measured the sequence quality according to five criteria: mismatch rate, gap rate, sequence precision, sequence recall, and edit distance, as they covered completeness and precision. SpecHLA outperformed HISAT-genotype to reconstruct sequences in 32 WGS samples from the HGSVC2 Project[18] (Figures 2F–2J). SpecHLA achieved average mismatch rate, gap rate, sequence precision, sequence recall, and edit distance of 2.99e−4, 1.75e−4, 98.8%, 94.3%, and 395 bp, respectively, while HISAT-genotype achieved 1.02e−3, 2.37e−3, 98.6%, 85.5%, and 985 bp, respectively. SpecHLA inferred entire HLA alleles with an average of three mismatches fewer, ten gaps fewer, and 590 bp less edit distance than HISAT-genotype per allele. SpecHLA reconstructed 71.1% more sequences than HISAT-genotype in all the loci (100% vs. 28.9%), highlighting SpecHLA's robustness (Figure 2K). Moreover, we compared the two methods for sequence reconstruction in the 2,000 simulated samples (Figures S4A–S4E). SpecHLA and HISAT-genotype had average mismatch rates of 1.96e−3 vs. 1.45e−2, gap rates of 5.14e−4 vs. 1.01e−2, sequence precisions of 93.3% vs. 87%, sequence recalls of 97.7% vs. 83%, and edit distances of 658 vs. 1,315 bp. SpecHLA was more accurate than HISAT-genotype across different sequencing settings. We

Figure 2. Validation of SpecHLA for 2-field and full-resolution HLA typing

(A–C) The 2-field typing accuracy in 230 WGS (A), 183 WES (B), and 462 RNA-seq (C) samples from the 1000 Genomes Project.

(D and E) The 2-field HLA typing accuracy of MHC class I (D) and MHC class II (E) genes with different sequencing depths in the IHWG cell lines subsampling dataset.

(F–K) The accuracy of inferring full-length HLA alleles with 32 HGSVC2 samples, measured by mismatch rate (F), gap rate (G), sequence precision (H), sequence recall (I), edit distance (J), and the number of samples with reconstructed sequences (K).

(L) The accuracy of G-group-resolution HLA typing on 32 HGSVC2 samples.

(M) The illustration of *HLA-A* genotypes phased by SpecHLA in two trios; each dot symbol represents a variant.

See also Figures S2–S4.

also applied SpecHLA and HISAT-genotype to recover novel alleles in the 50 simulated samples, and they achieved average mismatch rates of 1.74e−6 vs. 3.48e−4 and gap rates of 9.24e−6 vs. 2.59e−3 (Figures S2C–S2H). SpecHLA recovered simulated novel alleles more precisely than HISAT-genotype, suggesting that SpecHLA could reconstruct the HLA alleles absent from the database.

Also, SpecHLA inferred HLA exon sequences accurately in actual and simulated samples. In the HGSVC2 dataset, the G-group-resolution typing accuracies of SpecHLA, Kourami, HISAT-genotype, HLA-VBSeq, HLA*LA, and HLA-HD were 98.6% (505/512), 98.7% (379/384), 96.3% (493/512), 86.5% (443/512), 97.1% (497/512), and 94.7% (485/512), on average, respectively (Figure 2L). In the 2,000 simulated samples, SpecHLA yielded more accurate exon sequences of MHC class I genes than Kourami for all the datasets (Figures S4F and S4G), with average mismatch rates of 9.32e−4 vs. 6.05e−3 and gap rates of 3.30e−5 vs. 2.44e−3. SpecHLA was slightly less robust for MHC class II genes than Kourami in low-depth and short-read samples. With sequencing depth greater than 20× and reads longer than 90 bp, SpecHLA exhibited higher accuracies than Kourami, and they achieved average mismatch rates of 6.25e−4 vs. 2.04e−3 and gap rates of 7.35e−5 vs. 1.02e−3. Only exons 2 and 3 for MHC class I and only exon 2 for MHC class II genes were considered in the comparison.

Furthermore, SpecHLA reconstructed sequences highly trio consistent in two family trios. We calculated the difference between the child's inherited and the parent's original sequences. The difference was measured by mismatch rate and gap rate. On average, the mismatch and gap rates were 7.69e−4 and 2.63e−4, respectively (Figure S2I). We visualized the phased exonic variants of the trios using Oviz-Bio.[31] The phased genotypes were consistent with the trio structure (Figure 2M). Also, the 4-field typing results of SpecHLA were trio consistent for 93.8% (30/32) of the alleles in the children. These results consistently implied that SpecHLA is accurate in full-resolution HLA typing.

SpecHLA works with different data types

SpecHLA can integrate short and long reads for HLA typing. Seven individuals of HGSVC2 have both PE and PacBio data, and with these data, we performed SpecHLA using PE data and PE plus PacBio data separately. The mean mismatch rates without and with PacBio data were 1.95e−3 vs. 1.37e−3, and gap rates were 3.16e−4 vs. 2.98e−4 (Figures 3A and 3B). The result indicated that SpecHLA could efficiently integrate short and long reads to yield better results. SpecHLA can also accept long reads independently for HLA typing. Apart from the seven HGSVC2 PacBio samples, we collected a PacBio and an ONT sample of the individual NA12878, resulting in a total of nine long-read samples. In the nine long-read samples, SpecHLA and HLA*LA achieved G-group-resolution typing accuracies of 95.1% and 94.4%, respectively (Figure S2J).

In addition, simulated data showed that SpecHLA could combine PE data with PacBio, ONT, 10×, and Hi-C data. We generated 50 individuals using simulated novel alleles and produced sequencing reads with different protocols. We performed SpecHLA with different data permutations of the 50 individuals. The average mismatch rates were 5.84e−4, 5.46e−6, 8.92e−6, 2.61e−6, 2.61e−6, 2.03e−5, and 1.97e−4 using PE, PacBio, ONT, +PacBio, +ONT, +10×, and +Hi-C data, respectively (Figure 3C). The plus symbol (+) indicates the integration of PE and the present data. The highest mismatch rate was observed when utilizing only PE data. The lowest mismatch rate was achieved by integrating PE data with either PacBio or ONT data. Incorporating Hi-C data was not as efficient as long-read and 10× data.

SpecHLA could refine HLA typing with pedigree relations in actual and simulated family trios. We executed SpecHLA without and with pedigree relations in two family trios from the 1000 Genomes Project. The mean mismatch rates for children without and with pedigree relations were 1.10e−3 and 7.89e−4, respectively, while the corresponding gap rates were 3.39e−4 and 3.21e−4 (Figures 3D and 3E). We also simulated 50 family trios with PE reads; the read length, depth, and error rate were 75 bp, 30×, and 1%, respectively. In simulated trios, SpecHLA exhibited average mismatch rates of 3.59e−3 and 2.10e−3 for children without and with pedigree relations, respectively, and the corresponding gap rates were 9.06e−4 and 7.73e−4 (Figures S2K and S2L). The results indicated that SpecHLA reduced variant phasing ambiguities using pedigree relations.

SpecHLA demonstrated the ability to incorporate genotype frequency for HLA typing in allelic imbalance samples. We ran SpecHLA separately using only reads ("read"), using the integration of reads and genotype frequency ("hybrid"), and using only genotype frequency ("geno"). First, to investigate whether SpecHLA can incorporate genotype frequency, we performed SpecHLA on 200 allelic imbalance samples, which were LOH samples simulated from cancer cell lines. When solely using genotype frequency, SpecHLA achieved a 2-field typing accuracy of 90% (Figure 3F). Next, we examined the sequence reconstruction performance of SpecHLA after using genotype frequency. We ran SpecHLA in a simulated dataset comprising 80 allelic imbalance samples. Each gene contained one haplotype with a depth of 20× and the other haplotype with a depth of 80×. On average, in the dataset, the mismatch rates for "read," "hybrid," and "geno" were 3.49e−4, 3.98e−4, and 2.79e−4, respectively (Figure 3G). Notably, using only genotype frequency achieved the lowest mismatch rate. Furthermore, we tested SpecHLA's genotype frequency incorporation ability with different allelic depths. SpecHLA was applied to a dataset of 100 simulated novel-allele samples with varying allelic depths (Figure 3H). In this dataset, the average mismatch rates for "read," "hybrid," and "geno" were 9.93e−4, 4.34e−4, and 6.40e−4, respectively. The combined utilization of sequencing reads and genotype frequency achieved the best performance. These results showed that SpecHLA can efficiently utilize genotype frequency for HLA typing.

SpecHLA precisely infers HLA LOH events

SpecHLA identified HLA LOH events more accurately than DASH and LOHHLA in simulated data from cancer cell lines. We simulated 300 samples by combining HLA exonic data from cancer cell lines and matched normal cells, in which 200 samples possessed LOH events. For MHC class I genes, the LOH detection specificities of SpecHLA (99.7%) and LOHHLA

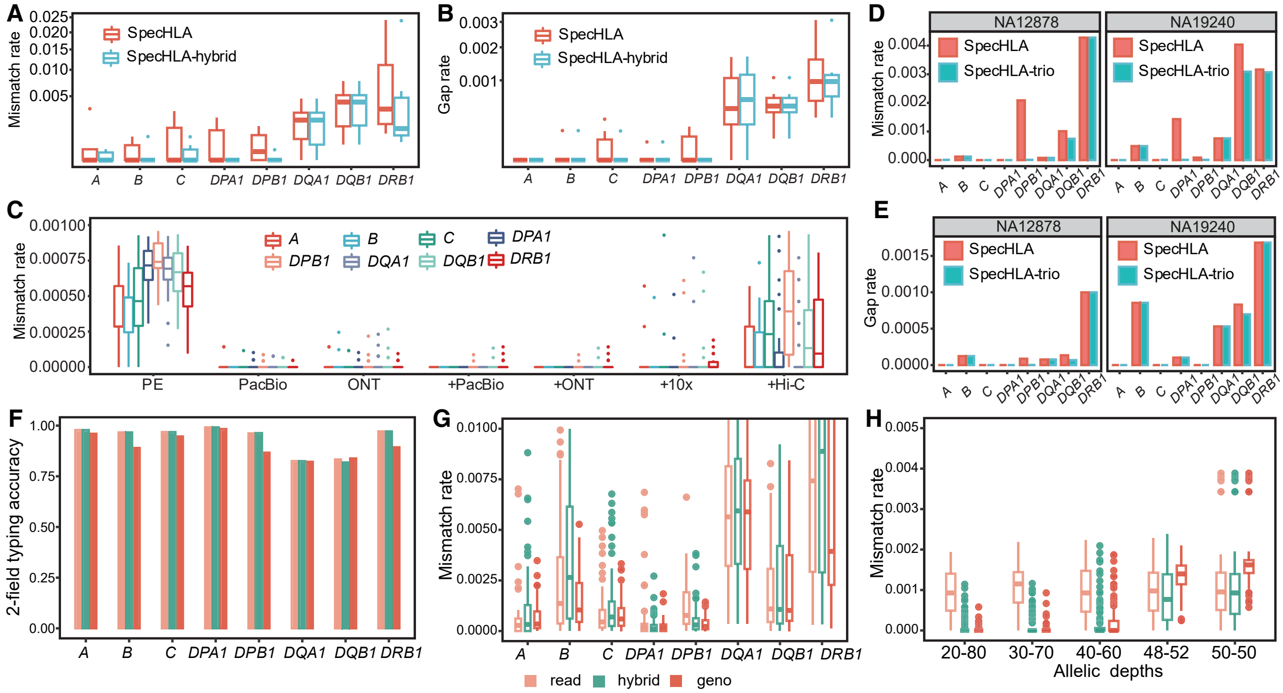

Figure 3. Validation of SpecHLA for using different data types

(A and B) Evaluation of the performance difference between SpecHLA with and without PacBio data across seven HGSVC2 individuals, quantified through the metrics of mismatch rate (A) and gap rate (B). "SpecHLA" indicates only using PE data, and "SpecHLA-hybrid" means integrating PE and PacBio data. The y axis is scaled using a square root transformation.

(C) The mismatch rate of SpecHLA with different data permutations of 50 simulated individuals. The plus symbol (+) indicates the integration of PE and the present data.

(D and E) The comparison of SpecHLA without and with pedigree relations in two family trios from the 1000 Genomes Project, measured by mismatch rate (D) and gap rate (E). "SpecHLA" indicates only using PE data, and "SpecHLA-trio" means integrating PE data and the pedigree relations.

(F–H) Evaluation of SpecHLA for incorporating genotype frequency. "Read" represents using only reads, "hybrid" represents using the integration of reads and genotype frequency, and "geno" represents using only genotype frequency.

(F) 2-field HLA typing accuracy in 200 LOH samples generated by cancer cell lines and normal cell data.

(G) Mismatch rate in 80 simulated allelic imbalance samples. In each sample, the depths of the two haplotypes are 20× and 80×.

(H) Mismatch rate of SpecHLA in 100 simulated novel-allele samples with different allelic depths, e.g., "30_70" indicates that the depths of the two haplotypes are 30× and 70×.

See also Figure S2.

(100.0%) were much higher than that of DASH (44.3%) (Figure 4). SpecHLA exhibited a higher sensitivity for LOH detection than LOHHLA across a range of tumor purities from 0.2 to 0.9, and the average sensitivities of SpecHLA and LOHHLA with tumor purity ranging from 0.1 to 1 were 96.8% and 92.8%, respectively. SpecHLA was more accurate than DASH and LOHHLA with MHC class I genes in the 300 samples. Additionally, SpecHLA exhibited a high LOH detection accuracy with a specificity of 97.5% and a sensitivity of 92.0% for MHC class II genes (Figure 4), but DASH and LOHHLA cannot handle such genes. Furthermore, through additional simulated data, we demonstrated the robustness of SpecHLA in handling allelic imbalance samples (Figure S2M) and different tumor purity and ploidy conditions (Figure S2N).

DISCUSSION

HLA genes persist in high sequence identity with the genes in the same class. For instance, *HLA-A* has high identities with *HLA-B*, *HLA-C*, and the pseudogene *HLA-Y*.[10] Reads from different HLA genes are mixed during sequencing; some of them are prone to align to the faulty gene, introducing errors in HLA typing. SpecHLA identifies the exact HLA locus for sequencing reads based on two assumptions: (1) HLA alleles belonging to the same HLA gene have a higher sequence identity than those from different genes, and (2) *de novo* alleles have high sequence identities with the known ones. SpecHLA permits adjusting read-assignment parameters to retain more novel small variants for *de novo* allele reconstruction. The curated HLA database grows rapidly.[29] SpecHLA has the advantage of adapting the iterative updates of the database. Based on the reads-binning strategy, we can use continuously added alleles in the database to assign reads more precisely. On the contrary, the database-matching methods optimized for a specific collection of alleles thus cannot efficiently remodel the ever-expanding list of alleles. Furthermore, they may even suffer from increased HLA alleles due to the increment of searching complexity.[14]

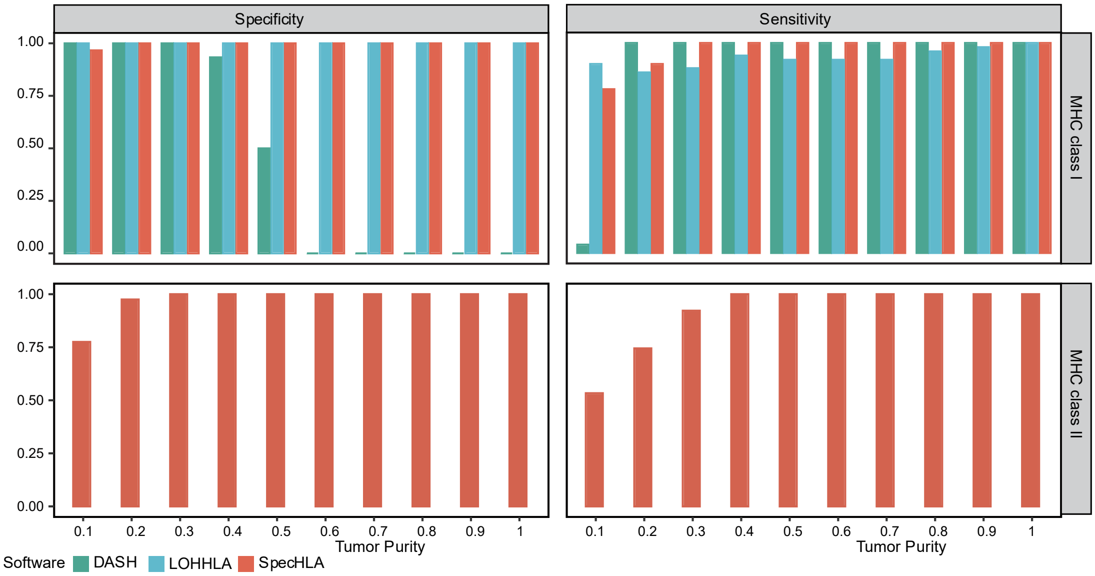

Figure 4. Validation of SpecHLA for HLA LOH detection in 300 simulated samples from cancer cell line data
The specificity and sensitivity of LOH detection were calculated separately for MHC class I and II genes. Only 200 out of the 300 samples harbored LOH events. Bar colors indicate different methods. DASH and LOHHLA are inapplicable for MHC class II genes.
See also Figure S2.

Previous research has highlighted the incompleteness of current HLA allele databases,[14] which poses a challenge for accurate HLA designation. Even if the reconstructed sequence is correct, the absence of the allele in the database can lead to erroneous designation results. Conversely, directly analyzing the HLA sequences is not affected by the incompleteness of the database. Furthermore, clustering the HLA sequences into groups using self-defined distance measurements has the potential to offer novel biological insights. As a result, we anticipate that conducting HLA typing at full resolution will become a prominent trend in future research endeavors.

MHC class II molecules present processed antigens to CD4(+) T lymphocytes, which is crucial for antigen-specific immune response.[32] Notably, LOH events can occur in MHC class II genes, as observed in a previous study on a patient with acute myeloid leukemia.[33] Tumor-specific MHC class II expression may be a clinically actionable biomarker of response to immune checkpoint inhibition, while LOH of MHC class II genes may reduce response to immunotherapies.[34] Many HLA-related tools merely focus on MHC class I genes. SpecHLA can perform full-resolution HLA typing and detect LOH events in MHC class II genes, which provides the opportunity to study MHC class II genes further.

Limitations of the study

To achieve accurate reconstruction of HLA sequences, our method, SpecHLA, requires a relatively high read alignment depth, typically 5× or greater. Any sequence regions with insufficient read depth are automatically masked by SpecHLA. Moreover, low-depth data can negatively impact the accuracy of SpecHLA's long indel detection, leading to lower completeness and increased contamination in the reconstructed HLA sequences.

STAR★METHODS

Detailed methods are provided in the online version of this paper and include the following:

- KEY RESOURCES TABLE
- RESOURCE AVAILABILITY
  - Lead contact
  - Materials availability
  - Data and code availability
- METHOD DETAILS
  - Algorithm of SpecHLA
  - Incorporating genotype frequency to phase in allelic imbalance samples
  - Benchmark datasets
  - Evaluation methods
- QUANTIFICATION AND STATISTICAL ANALYSIS

SUPPLEMENTAL INFORMATION

Supplemental information can be found online at https://doi.org/10.1016/j.crmeth.2023.100589.

ACKNOWLEDGMENTS

This work described in this paper is funded by the Applied Research Grant 9667204. We thank Prof. Sean Michael Boyle for providing the CRL-2314, CRL-5915, and CRL-5922 cell lines data and matched normal cell data. We also thank Mr. Yonghan Yu for the assistance in using SpecHap and thank Mr. Bowen Tan for the help in the long indel phasing.

AUTHOR CONTRIBUTIONS

S.C.L. designed and supervised the study. S.W. and M.W. developed and evaluated the software. L.C. was involved in software implementation. G.P.

CellPress
OPEN ACCESS

contributed to pedigree integration. M.W. and Y.W. were engaged in figure design. S.W. and M.W. wrote the manuscript, and all authors contributed to manuscript revisions.

DECLARATION OF INTERESTS

The authors declare no competing interests.

DECLARATION OF GENERATIVE AI AND AI-ASSISTED TECHNOLOGIES IN THE WRITING PROCESS

During the preparation of this work, the authors used ChatGPT to refine English sentences. After using this tool, the authors reviewed and edited the content as necessary and take full responsibility for the publication's content.

Received: January 11, 2023
Revised: June 20, 2023
Accepted: August 21, 2023
Published: September 14, 2023

REFERENCES

1. Benacerraf, B. (1981). Role of MHC gene products in immune regulation. Science *212*, 1229–1238. https://doi.org/10.1126/science.6165083.
2. Opelz, G., Wujciak, T., Döhler, B., Scherer, S., and Mytilineos, J. (1999). HLA compatibility and organ transplant survival. Rev. Immunogenet. *1*, 334–342.
3. Badrising, U.A., Schreuder, G.M.T., Giphart, M.J., Geleijns, K., Verschuuren, J.J.G.M., Wintzen, A.R., Maat-Schieman, M.L.C., van Doorn, P., van Engelen, B.G.M., Faber, C.G., et al. (2004). Associations with autoimmune disorders and HLA class I and II antigens in inclusion body myositis. Neurology *63*, 2396–2398. https://doi.org/10.1212/01.wnl.0000148588.15052.4c.
4. Blackwell, J.M., Jamieson, S.E., and Burgner, D. (2009). HLA and infectious diseases. Clin. Microbiol. Rev. *22*, 370–385, Table of Contents. https://doi.org/10.1128/cmr.00048-08.
5. Stransky, N., Egloff, A.M., Tward, A.D., Kostic, A.D., Cibulskis, K., Sivachenko, A., Kryukov, G.V., Lawrence, M.S., Sougnez, C., McKenna, A., et al. (2011). The mutational landscape of head and neck squamous cell carcinoma. Science *333*, 1157–1160. https://doi.org/10.1126/science.1208130.
6. Lee, H., and Kingsford, C. (2018). Kourami: graph-guided assembly for novel human leukocyte antigen allele discovery. Genome Biol. *19*, 16. https://doi.org/10.1186/s13059-018-1388-2.
7. Szolek, A., Schubert, B., Mohr, C., Sturm, M., Feldhahn, M., and Kohlbacher, O. (2014). OptiType: precision HLA typing from next-generation sequencing data. Bioinformatics *30*, 3310–3316. https://doi.org/10.1093/bioinformatics/btu548.
8. Shukla, S.A., Rooney, M.S., Rajasagi, M., Tiao, G., Dixon, P.M., Lawrence, M.S., Stevens, J., Lane, W.J., Dellagatta, J.L., Steelman, S., et al. (2015). Comprehensive analysis of cancer-associated somatic mutations in class I HLA genes. Nat. Biotechnol. *33*, 1152–1158. https://doi.org/10.1038/nbt.3344.
9. Nariai, N., Kojima, K., Saito, S., Mimori, T., Sato, Y., Kawai, Y., Yamaguchi-Kabata, Y., Yasuda, J., and Nagasaki, M. (2015). HLA-VBSeq: accurate HLA typing at full resolution from whole-genome sequencing data. BMC Genom. *16*, S7. https://doi.org/10.1186/1471-2164-16-S2-S7.
10. Kawaguchi, S., Higasa, K., Shimizu, M., Yamada, R., and Matsuda, F. (2017). HLA-HD: An accurate HLA typing algorithm for next-generation sequencing data. Hum. Mutat. *38*, 788–797. https://doi.org/10.1002/humu.23230.
11. Dilthey, A.T., Gourraud, P.A., Mentzer, A.J., Cereb, N., Iqbal, Z., and McVean, G. (2016). High-accuracy HLA type inference from whole-genome sequencing data using population reference graphs. PLoS Comput. Biol. *12*, e1005151. https://doi.org/10.1371/journal.pcbi.1005151.
12. Orenbuch, R., Filip, I., Comito, D., Shaman, J., Pe'er, I., and Rabadan, R. (2020). arcasHLA: high-resolution HLA typing from RNAseq. Bioinformatics *36*, 33–40. https://doi.org/10.1093/bioinformatics/btz474.
13. Dilthey, A.T., Mentzer, A.J., Carapito, R., Cutland, C., Cereb, N., Madhi, S.A., Rhie, A., Koren, S., Bahram, S., McVean, G., and Phillippy, A.M. (2019). HLA* LA—HLA typing from linearly projected graph alignments. Bioinformatics *35*, 4394–4396. https://doi.org/10.1093/bioinformatics/btz235.
14. Matey-Hernandez, M.L., Danish Pan Genome Consortium; Brunak, S., and Izarzugaza, J.M.G. (2018). Benchmarking the HLA typing performance of Polysolver and Optitype in 50 Danish parental trios. BMC Bioinf. *19*, 239. https://doi.org/10.1186/s12859-018-2239-6.
15. Kim, D., Paggi, J.M., Park, C., Bennett, C., and Salzberg, S.L. (2019). Graph-based genome alignment and genotyping with HISAT2 and HISAT-genotype. Nat. Biotechnol. *37*, 907–915. https://doi.org/10.1038/s41587-019-0201-4.
16. Hayashi, S., Yamaguchi, R., Mizuno, S., Komura, M., Miyano, S., Nakagawa, H., and Imoto, S. (2018). ALPHLARD: a Bayesian method for analyzing HLA genes from whole genome sequence data. BMC Genom. *19*, 790–811. https://doi.org/10.1186/s12864-018-5169-9.
17. Chin, C.S., Wagner, J., Zeng, Q., Garrison, E., Garg, S., Fungtammasan, A., Rautiainen, M., Aganezov, S., Kirsche, M., Zarate, S., et al. (2020). A diploid assembly-based benchmark for variants in the major histocompatibility complex. Nat. Commun. *11*, 4794–4799. https://doi.org/10.1038/s41467-020-18564-9.
18. Ebert, P., Audano, P.A., Zhu, Q., Rodriguez-Martin, B., Porubsky, D., Bonder, M.J., Sulovari, A., Ebler, J., Zhou, W., Serra Mari, R., et al. (2021). Haplotype-resolved diverse human genomes and integrated analysis of structural variation. Science *372*, eabf7117. https://doi.org/10.1126/science.abf7117.
19. Zheng, G.X.Y., Lau, B.T., Schnall-Levin, M., Jarosz, M., Bell, J.M., Hindson, C.M., Kyriazopoulou-Panagiotopoulou, S., Masquelier, D.A., Merrill, L., Terry, J.M., et al. (2016). Haplotyping germline and cancer genomes with high-throughput linked-read sequencing. Nat. Biotechnol. *34*, 303–311. https://doi.org/10.1038/nbt.3432.
20. Kaplan, N., and Dekker, J. (2013). High-throughput genome scaffolding from in vivo DNA interaction frequency. Nat. Biotechnol. *31*, 1143–1147. https://doi.org/10.1038/nbt.2768.
21. Amarasinghe, S.L., Su, S., Dong, X., Zappia, L., Ritchie, M.E., and Gouil, Q. (2020). Opportunities and challenges in long-read sequencing data analysis. Genome Biol. *21*, 30. https://doi.org/10.1186/s13059-020-1935-5.
22. Garg, S., Aach, J., Li, H., Sebenius, I., Durbin, R., and Church, G. (2020). A haplotype-aware de novo assembly of related individuals using pedigree sequence graph. Bioinformatics *36*, 2385–2392. https://doi.org/10.1093/bioinformatics/btz942.
23. Wang, S., Jiang, Y., and Li, S. (2021). PStrain: An Iterative Microbial Strains Profiling Algorithm for Shotgun Metagenomic Sequencing Data. Bioinformatics *36*, 5499–5506. https://doi.org/10.1093/bioinformatics/btaa1056.
24. Montesion, M., Murugesan, K., Jin, D.X., Sharaf, R., Sanchez, N., Guria, A., Minker, M., Li, G., Fisher, V., Sokol, E.S., et al. (2021). Somatic HLA class I loss is a widespread mechanism of immune evasion which refines the use of tumor mutational burden as a biomarker of checkpoint inhibitor response. Cancer Discov. *11*, 282–292. https://doi.org/10.1158/2159-8290.cd-20-0672.
25. Tran, E., Robbins, P.F., Lu, Y.C., Prickett, T.D., Gartner, J.J., Jia, L., Pasetto, A., Zheng, Z., Ray, S., Groh, E.M., et al. (2016). T-cell transfer therapy targeting mutant KRAS in cancer. N. Engl. J. Med. *375*, 2255–2262. https://doi.org/10.1056/nejmoa1609279.
26. McGranahan, N., Rosenthal, R., Hiley, C.T., Rowan, A.J., Watkins, T.B.K., Wilson, G.A., Birkbak, N.J., Veeriah, S., Van Loo, P., Herrero, J., et al. (2017). Allele-specific HLA loss and immune escape in lung cancer evolution. Cell *171*, 1259–1271.e11. https://doi.org/10.1016/j.cell.2017.10.001.

27. Pyke, R.M., Mellacheruvu, D., Dea, S., Abbott, C.W., McDaniel, L., Bhave, D.P., Zhang, S.V., Levy, E., Bartha, G., West, J., et al. (2022). A machine learning algorithm with subclonal sensitivity reveals widespread pan-cancer human leukocyte antigen loss of heterozygosity. Nat. Commun. *13*, 1925–2013. https://doi.org/10.1038/s41467-022-29203-w.

28. Yu, Y., Chen, L., Miao, X., and Li, S.C. (2021). SpecHap: a diploid phasing algorithm based on spectral graph theory. Nucleic Acids Res. *49*, e114. https://doi.org/10.1093/nar/gkab709.

29. Robinson, J., Halliwell, J.A., Hayhurst, J.D., Flicek, P., Parham, P., and Marsh, S.G.E. (2015). The IPD and IMGT/HLA database: allele variant databases. Nucleic Acids Res. *43*, D423–D431. https://doi.org/10.1093/nar/gku1161.

30. Norman, P.J., Norberg, S.J., Guethlein, L.A., Nemat-Gorgani, N., Royce, T., Wroblewski, E.E., Dunn, T., Mann, T., Alicata, C., Hollenbach, J.A., et al. (2017). Sequences of 95 human MHC haplotypes reveal extreme coding variation in genes other than highly polymorphic HLA class I and II. Genome Res. *27*, 813–823. https://doi.org/10.1101/gr.213538.116.

31. Jia, W., Li, H., Li, S., Chen, L., and Li, S.C. (2020). Oviz-Bio: a web-based platform for interactive cancer genomics data visualization. Nucleic Acids Res. *48*, W415–W426. https://doi.org/10.1093/nar/gkaa371.

32. Holling, T.M., Schooten, E., and van Den Elsen, P.J. (2004). Function and regulation of MHC class II molecules in T-lymphocytes: of mice and men. Hum. Immunol. *65*, 282–290. https://doi.org/10.1016/j.humimm.2004.01.005.

33. Lobashevsky, A.L., Krueger-Sersen, M., Britton, R.M., Littrell, C.A., Singh, S., Cui, C.P., Kashi, Z., Martin, R.K., Breman, A.M., Vance, G.H., and Farag, S.S. (2019). Pretransplant HLA typing revealed loss of heterozygosity in the major histocompatibility complex in a patient with acute myeloid leukemia. Hum. Immunol. *80*, 257–262. https://doi.org/10.1016/j.humimm.2019.02.009.

34. Axelrod, M.L., Cook, R.S., Johnson, D.B., and Balko, J.M. (2019). Biological consequences of MHC-II expression by tumor cells in cancer. Clin. Cancer Res. *25*, 2392–2402. https://doi.org/10.1158/1078-0432.ccr-18-3200.

35. Li, H. (2013). Aligning sequence reads, clone sequences and assembly contigs with BWA-MEM. Preprint at arXiv. https://doi.org/10.1048550/arXiv.1303.3997.

36. Altschul, S.F., Madden, T.L., Schäffer, A.A., Zhang, J., Zhang, Z., Miller, W., and Lipman, D.J. (1997). Gapped BLAST and PSI-BLAST: a new generation of protein database search programs. Nucleic Acids Res. *25*, 3389–3402. https://doi.org/10.1093/nar/25.17.3389.

37. Langmead, B., and Salzberg, S.L. (2012). Fast gapped-read alignment with Bowtie 2. Nat. Methods *9*, 357–359. https://doi.org/10.1038/nmeth.1923.

38. Yang, R., Nelson, A.C., Henzler, C., Thyagarajan, B., and Silverstein, K.A.T. (2015). ScanIndel: a hybrid framework for indel detection via gapped alignment, split reads and de novo assembly. Genome Med. *7*, 127–212. https://doi.org/10.1186/s13073-015-0251-2.

39. Li, H. (2015). FermiKit: assembly-based variant calling for Illumina resequencing data. Bioinformatics *31*, 3694–3696. https://doi.org/10.1093/bioinformatics/btv440.

40. Carter, S.L., Cibulskis, K., Helman, E., McKenna, A., Shen, H., Zack, T., Laird, P.W., Onofrio, R.C., Winckler, W., Weir, B.A., et al. (2012). Absolute quantification of somatic DNA alterations in human cancer. Nat. Biotechnol. *30*, 413–421. https://doi.org/10.1038/nbt.2203.

41. Luo, R., Sedlazeck, F.J., Darby, C.A., Kelly, S.M., and Schatz, M.C. (2017). LRSim: a linked-reads simulator generating insights for better genome partitioning. Comput. Struct. Biotechnol. J. *15*, 478–484. https://doi.org/10.1016/j.csbj.2017.10.002.

42. Li, H. (2018). Minimap2: pairwise alignment for nucleotide sequences. Bioinformatics *34*, 3094–3100. https://doi.org/10.1093/bioinformatics/bty191.

43. Chen, S., Zhou, Y., Chen, Y., and Gu, J. (2018). fastp: an ultra-fast all-in-one FASTQ preprocessor. Bioinformatics *34*, i884–i890. https://doi.org/10.1093/bioinformatics/bty560.

44. Ono, Y., Asai, K., and Hamada, M. (2013). PBSIM: PacBio reads simulator—toward accurate genome assembly. Bioinformatics *29*, 119–121. https://doi.org/10.1093/bioinformatics/bts649.

45. Yang, C., Chu, J., Warren, R.L., and Birol, I. (2017). NanoSim: nanopore sequence read simulator based on statistical characterization. GigaScience *6*, 1–6. https://doi.org/10.1093/gigascience/gix010.

46. DeMaere, M.Z., and Darling, A.E. (2018). Sim3C: simulation of Hi-C and Meta3C proximity ligation sequencing technologies. GigaScience *7*, 1–12. https://doi.org/10.1093/gigascience/gix103.

47. Edge, P., and Bansal, V. (2019). Longshot enables accurate variant calling in diploid genomes from single-molecule long read sequencing. Nat. Commun. *10*, 4660–4710. https://doi.org/10.1038/s41467-019-12493-y.

48. Danecek, P., and McCarthy, S.A. (2017). BCFtools/csq: haplotype-aware variant consequences. Bioinformatics *33*, 2037–2039. https://doi.org/10.1093/bioinformatics/btx100.

49. Garrison, E., and Marth, G. (2012). Haplotype-based variant detection from short-read sequencing. Preprint at arXiv. https://doi.org/10.1048550/arXiv.1207.3907.

STAR★METHODS

KEY RESOURCES TABLE

REAGENT or RESOURCE	SOURCE	IDENTIFIER
Deposited data		
WGS data for 2-field resolution typing evaluation	1000 Genome project	http://ftp.1000genomes.ebi.ac.uk/vol1/ftp/data_collections/1000G_2504_high_coverage/1000G_2504_high_coverage.sequence.index; RRID: SCR_006828
WES data for 2-field resolution typing evaluation	1000 Genome project	http://ftp.1000genomes.ebi.ac.uk/vol1/ftp/data_collections/1000_genomes_project/data/; RRID: SCR_006828
RNA-seq data for 2-field resolution typing evaluation	1000 Genome project	https://www.ebi.ac.uk/arrayexpress/experiments/E-GEUV-1/samples/; RRID: SCR_006828
HLA type data of 1000 Genome samples used as ground truth	1000 Genome project	http://ftp.1000genomes.ebi.ac.uk/vol1/ftp/data_collections/HLA_types/20181129_HLA_types_full_1000_Genomes_Project_panel.txt; RRID: SCR_006828
A Panel of IHWG Lymphoblastoid B Cell Lines	NCBI	https://www.ncbi.nlm.nih.gov/pmc/articles/PMC5411776/; BioProject: PRJEB6763; RRID: SCR_004871
HGSVC2 data for full-resolution typing evaluation	HGSVC2 project	https://www.internationalgenome.org/data-portal/data-collection/hgsvc2
DASH HLA LOH cancer cell lines	Pyke et al.[27]	https://www.nature.com/articles/s41467-022-29203-w
LOH results of DASH data as ground truth	Pyke et al.[27]	https://static-content.springer.com/esm/art%3A10.1038%2Fs41467-022-29203-w/MediaObjects/41467_2022_29203_MOESM1_ESM.docx
NA12878 PacBio data	NCBI	SRA:SRR3197748; RRID: SCR_004891
NA12878 ONT data	GIAB	http://ftp-trace.ncbi.nlm.nih.gov/ReferenceSamples/giab/data/NA12878/Ultralong_OxfordNanopore/NA12878-minion-ul_GRCh38.bam
Software and algorithms		
SpecHLA v1.0.1	This paper	https://doi.org/10.5281/zenodo.8232273
BWA-0.7.17-r1188	Li[35]	https://github.com/lh3/bwa
blast 2.12.0	Altschul et al.[36]	https://blast.ncbi.nlm.nih.gov/Blast.cgi
Novoalign V4.02.01	NOVOCRAFT	https://www.novocraft.com/products/novoalign/
Bowtie2 v2.3.4.1	Langmead and Salzberg[37]	https://github.com/BenLangmead/bowtie2
ScanIndel v1.3	Yang et al.[38]	https://github.com/cauyrd/ScanIndel
Fermikit-0.13	Li[39]	https://github.com/lh3/fermikit
SpecHap v1.0.1	Yu et al.[28]	https://github.com/deepomicslab/SpecHap
arcasHLA v0.5.0	Orenbuch et al.[12]	https://github.com/RabadanLab/arcasHLA
HISAT-genotype v1.3.2	Kim et al.[15]	https://github.com/DaehwanKimLab/hisat-genotype
HLA-HD v1.4.0	Kawaguchi et al.[10]	https://www.genome.med.kyoto-u.ac.jp/HLA-HD/
HLA*LA v1.0.2	Dilthey et al.[13]	https://github.com/DiltheyLab/HLA-LA
HLA-VBseq v2	Nariai et al.[9]	http://nagasakilab.csml.org/hla/
Kourami v0.9.6	Lee and Kingsford[6]	https://github.com/Kingsford-Group/kourami
OptiType v1.3.1	Szolek et al.[7]	https://github.com/FRED-2/OptiType
PolySolver v1.0.0	Shukla et al.[8]	https://github.com/jason-weirather/hla-polysolver
DASH training.xgboost_model.2021_05_10.p	Pyke et al.[27]	https://github.com/Personalis-DASH/DASH
LOHHLA v1.1.6	McGranahan et al.[26]	https://github.com/mskcc/lohhla
ABSOLUTE v1.2	Carter et al.[40]	https://bioinformaticshome.com/tools/cnv/descriptions/ABSOLUTE.html#gsc.tab=0
pbsv Version 2.6.2	PacificBiosciences	https://github.com/PacificBiosciences/pbbioconda
pbmm2-1.4.0	PacificBiosciences	https://github.com/PacificBiosciences/pbbioconda
LRSIM v1.0	Luo et al.[41]	https://github.com/aquaskyline/LRSIM
LongRanger v2.2.2	10x Genomics	https://support.10xgenomics.com/genome-exome/software/downloads/latest

(Continued on next page)

Continued		
REAGENT or RESOURCE	SOURCE	IDENTIFIER
Minimap 2.17-r941	Li[42]	https://github.com/lh3/minimap2
fastp 0.20.0	Chen et al.[43]	https://github.com/OpenGene/fastp
PBSIM v1.0.3	Ono et al.[44]	https://doi.org/10.1093/bioinformatics/bts649
NanoSim v3.1.0	Yang et al.[45]	https://github.com/bcgsc/NanoSim
sim3C v0.2	DeMaere and Darling[46]	https://github.com/cerebis/sim3C
DWGSIM v0.1.13	GitHub	https://github.com/nh13/DWGSIM
Longshot v0.4.1	Edge and Bansal[47]	https://github.com/pjedge/longshot
bcftools-Version: 1.9	Danecek and McCarthy[48]	http://www.htslib.org/doc/1.0/bcftools.html
Oviz-bio	Jia et al.[31]	https://bio.oviz.org/

RESOURCE AVAILABILITY

Lead contact

Further information and requests for resources and reagents should be directed to and will be fulfilled by the lead contact, Shuai Cheng Li (shuaicli@cityu.edu.hk).

Materials availability

This study did not generate new unique reagents.

Data and code availability

- This paper analyzes existing, publicly available data. The accession numbers for the datasets are listed in the key resources table.
- The software package SpecHLA is publicly available at https://github.com/deepomicslab/SpecHLA. Detailed benchmark experiment results have also been deposited in this repository. Additionally, the original source code has been deposited at Zenodo and is publicly available as of the date of publication. DOIs are listed in the key resources table.
- Any additional information required to reanalyze the data reported in this paper is available from the lead contact upon request.

METHOD DETAILS

Algorithm of SpecHLA

Binning reads

SpecHLA employs a read-binning procedure to reduce the ambiguity of HLA read alignment. We conduct read binning by assigning reads to HLA loci based on their sequence identity to existing HLA alleles. A lite version of IMGT/HLA database[29] (Release 3.37.0) is constructed with 6,172 alleles of 39 HLA genes and pseudogenes (Table S1). Given a sample, we first collect the reads mapped to the HLA region with a method similar to Kourami[6] and align them to the lite IMGT/HLA database using Novoalign v.4.2.1 (http://www.novocraft.com/products/novoalign/). In the absence of the Novoalign license, Bowtie2 v2.3.4.1[37] is offered as an alternative. Each read is then assigned to its best-matched allele, that is, the allele with the least number of mismatches with the read. Then a PE read is assigned to an HLA locus to which the best-matched allele belongs if it satisfies the four additional criteria: (i) Both its two ends are aligned to the same best-matched allele. (ii) The number of mismatches between each end and the best-matched allele is no more than θ (θ is 2 by default). (iii) The sequence identity between the read and the best-matched allele is at least υ higher than the sequence identity between the read and the alleles of any other HLA loci. By default, υ is 0.1. (iv) The read is aligned to the best-matched allele without soft-clipping. The accuracy of read binning with different θ values is tabulated in Figure S1H-M. In 50 simulated samples, we validated SpecHLA's ability to accurately type alleles that are not included in the lite IMGT/HLA database, achieving a 2-field typing accuracy of 93.38%.

Mapping reads to the reference

SpecHLA further utilizes local assembly to align the binned reads to the reference more accurately. Alignment algorithms rely on scoring systems. If the reads from alleles heavily diverge from the reference, homologous reads might be aligned to alternative coordinates on the reference. First, we construct the *IMGT representative reference* by concatenating the first recorded allele at each HLA locus from the IMGT/HLA database (Table S1). The HLA locus may contain some *highly divergent regions* on the reference (The divergent regions are selected empirically and listed in Table S1). Then, at each HLA locus, we map the binned reads to the IMGT representative reference (hereafter, IMGT reference) using BWA MEM v.0.7.17.[35] Next, local assembly is performed to rectify the read mapping in highly divergent regions; we extract the reads mapped to each highly divergent region and independently assemble

them into contigs with Fermikit v.0.13.[39] We may have one or more assembled contigs for each highly divergent region. After that, each extracted read is then mapped to the assembled contig with the highest alignment score by BWA MEM. We also map the contigs to the IMGT reference using Blastn v.2.3.0.[36] Last, we re-build the alignment between the extracted reads and the IMGT reference by projecting the reads onto the IMGT reference according to aligned loci between the reads and the contig, and between the contig and the IMGT reference.

Calling variants

With the reads aligned to the IMGT reference, we identify the variants. We categorized the variants into two types, *small variants* and long indels. Small variants consist of single nucleotide variants (SNVs) and short indels. Long indels are insertions and deletions longer than 150 bp. We detect both small variants and long indels from the reads alignment. Freebayes v.1.2.0[49] is used to call small variants, and ScanIndel v.1.3[38] is adopted to detect long indels. To handle PacBio reads, we also allow adopting pbsv v.2.6.2 to call long indels based on the alignment file processed by pbmm2 v.1.4.0 (https://github.com/PacificBiosciences/pbbioconda). At the variant locus with more than two alleles, if the sum of the frequency of the two highest-frequency alleles is larger than 0.7, we retain the two highest-frequency alleles of the variant. Otherwise, we discard the variant.

Phasing small variants guided by spectral graph theory

SpecHLA phases small variants based on spectral graph theory using SpecHap v.1.0.[28] We model the variant phasing problem as a graph-bipartition problem. With n heterozygous variants, we construct an undirected graph G of $2n$ vertices. Assume each locus has two variants. Each vertex represents an allele of the variant locus (0 or 1). The edge among two vertices of different loci indicates the two variants are from the same haplotype; the edge weight is the logarithmic likelihood of the corresponding haplotype.

We designate reads that have been mapped to a minimum of two variant loci as *phase-informative reads*. These reads can originate from a range of sequencing protocols, including but not limited to PE, 10x, Hi-C, PacBio, and ONT. We calculate the edge weight from phase-informative reads. Assume $q_{i,j}$ as the likelihood of nucleotide(s) at variant locus j is mistaken on read R_i. At two variant loci, given the haplotype h, the likelihood of observing the read R_i is as

$$p(h) = \prod_j (1 - q_{i,j})_{R_{i,j} = h_j} \prod_j (q_{i,j})_{R_{i,j} \neq h_j}, \quad \text{(Equation 1)}$$

where $(1 - q_{i,j})_{R_{i,j} = h_j}$ is $(1 - q_{i,j})$ if $R_{i,j}$ and h_j are equal, and 1 otherwise; $(q_{i,j})_{R_{i,j} \neq h_j}$ is $(q_{i,j})$ if $R_{i,j}$ and h_j are different, and 1 otherwise. Denote $\overline{h}$ as the complementary haplotype of h, the likelihood of the read R_i derives from $\overline{h}$ is $p(\overline{h})$. We determine which haplotype the read supports by comparing the likelihoods with $\max\{p(h), p(\overline{h})\}$. Given the self-complement haplotype pair $H = (h, \overline{h})$, the likelihood of observing the read set R is inferred as

$$p(H) = \prod_i \max\{p(h), p(\overline{h})\}, \quad \text{(Equation 2)}$$

where i indicates the index of the read in the set R. Then, we assume the edge weight of conflicting haplotype H_1 and H_2 as

$$E_{H_1} = \max\left\{\log\frac{p(H_1)}{p(H_2)}, 0\right\}, \quad \text{(Equation 3)}$$

$$E_{H_2} = \max\left\{\log\frac{p(H_2)}{p(H_1)}, 0\right\}. \quad \text{(Equation 4)}$$

In this way, we deduce the edge weight between every two variant loci. After the graph construction, the $2n$ vertices of the graph G can be partitioned into two subgroups by the sign (+/−) of the Fiedler vector. Therefore, we obtain the haplotypes equivalent to vertex subgroups. The locus with the absolute value of the Fiedler vector lower than 1e-5 will not be phased. We illustrate an example of phasing variants based on the Fiedler vector in Figure S1A.

Incorporating genotype frequency to phase in allelic imbalance samples

The alleles from different variant loci on the same haplotype should share similar frequencies. Hence, genotype frequency can guide variant phasing in allelic imbalance samples.[23] SpecHLA incorporates genotype frequencies in edge weight computation. Assume $\beta(h_j)$ as the frequency of allele h_j at variant locus j. At two variant loci, given a self-complement haplotype pair $H = (h, \overline{h})$, we define the function f as

$$f(H) = \max\left\{\prod_j \beta(h_j), \prod_j \beta(\overline{h}_j)\right\}. \quad \text{(Equation 5)}$$

Given conflicting haplotype H_1 and H_2, we calculate the edge weight from both sequencing reads and genotype frequency as

$$G_{H_1} = (1 - w)E_{H_1} + w\max\left\{\log\frac{f(H_1)}{f(H_2)}, 0\right\}, \quad \text{(Equation 6)}$$

$$G_{H_2} = (1 - w)E_{H_2} + w \max\left\{\log\frac{f(H_2)}{f(H_1)}, 0\right\}, \quad \text{(Equation 7)}$$

where w is hyper-parameter, $0 \le w \le 1$, w is 0 by default. The genotype frequency is incorporated into variant linkage graph construction for variant phasing. Evaluation of SpecHLA with different values of w can be seen in Figures S2O–S2Q.

Adopting pedigree relations to phase

SpecHLA has the capability to enhance phasing results through the utilization of pedigree relations when such relations are accessible. We refine the phasing outcome by establishing a connection between the child's unlinked blocks guided by the parents' phased blocks. Moreover, at each phased block of the child, we employ parental locus linkage as a reference, enabling the correction of the child's haplotype in instances where conflicts arise.

Specifically, let S represent the child's phased blocks, F denote the father's phased blocks, and M denote the mother's phased blocks. SpecHLA identifies connections between distinct phased blocks of the child based on parental blocks. Let S_i and S_j represent two unconnected phased blocks of the child. If a parental block ϕ ($\phi \in F \cup M$) encompasses both blocks, we consider a link to exist between S_i and S_j. Two types of links may exist: (i) direct link from S_i to S_j, and (ii) link from S_i to the flipped version of S_j. If the parental blocks support different link types, SpecHLA assigns a score ϖ to each link type and determines the final link type with a higher ϖ value. The calculation of ϖ involves examining the variant sites in the overlap region of ϕ with S_i and S_j separately. Consider one haplotype of a parental block ϕ. If the alleles from the child and parents are the same at a site, ϖ is incremented by one; otherwise, it is decremented by one. This process is repeated for the other haplotype of ϕ (i.e., the complement haplotype), and the maximum value is selected as the final ϖ score.

SpecHLA can also correct falsely phased variants of children using parental phased blocks. If a variant within the child-phased block conflicts with both the paternal and maternal haplotypes, SpecHLA identifies this site as wrongly phased and flips it. For instance, consider a child's phased block with two variant sites: the first haplotype is [0,1], and the other haplotype is [1,0]. If the haplotype of the father is [0,0] and the haplotype of the mother is [1,1], SpecHLA will flip one of the variant sites in the child.

Phasing unlinked blocks guided by HLA database

SpecHLA uses known HLA alleles from the IMGT/HLA database to phase unlinked blocks based on spectral graph theory. The procedure is based on the assumption that a haplotype with higher similarity to known HLA alleles is more likely to be correct. To construct the linkage graph, we denote the self-complement haplotype pair of each phased block as two vertices. The edge indicates that the haplotypes should be linked. We merge the haplotypes from two blocks and align the merged haplotype h to known HLA alleles. For each mapped allele, we calculate an alignment score γ according to the mapping identity I and the mapped length L by $\gamma = IL$. $\delta(h)$ represents the highest γ value among the mapped alleles of h. At two blocks, for a self-complement merged haplotype pair $H = (h, \overline{h})$, we have

$$\delta(H) = \delta(h) + \delta(\overline{h}). \quad \text{(Equation 8)}$$

The edge weight of conflicting haplotype H_1 and H_2 is defined as $\delta(H_1)$ and $\delta(H_2)$, respectively. Afterward, unlinked blocks are phased by partitioning the vertices with the Fiedler vector.

Phasing long indel loci

We link the long indel to the small variant phased haplotypes. The long insertion sequences and the IMGT representative reference are combined to generate a modified reference. The modified reference is split into segments based on the breakpoints of long indels. In particular, if a deletion overlaps with another, it would be divided into two segments at the overlap boundary. The segments can be classified into three categories: insertion, deletion, and regular. We align reads to the modified reference using BWA MEM. The copy number of long indels is estimated based on the reads alignment. We compute the average depth of all regular segments (μ) and each insertion segment (σ). The copy number of the insertion segment is deduced as two if $\sigma/\mu > 0.85$ and one otherwise. Phasing of small variants takes place within insertion segments characterized by a copy number of two. For each deletion segment, the unmapped ratio (representing the proportion of zero-depth regions) is assessed. A copy number of zero is inferred for deletion segments if the unmapped ratio exceeds 0.2; otherwise, it is determined as one.

The heterozygous long indels are phased with the supporting reads. This involves the collection of reads that independently support each haplotype phased for small variants. Additionally, the determination of which allele is supported by the reads at each long indel locus allows for a linkage to a haplotype featuring a greater number of shared supporting reads. The diploid sequences are generated with phased small variants and long indels. Finally, we slide a window of 20 bp along the gene; the window with a mean depth less than ζ is masked with 'N' (ζ is five by default).

Sequence inference of the HLA-DRB1 duplication

The region spanning 3,900 to 4,400 bp within the *HLA-DRB1* locus harbors a long duplication in the IMGT representative reference. In response to this challenge, a strategic approach was implemented: homologous sequences of this region were extracted from the entire ensemble of *HLA-DRB1* alleles contained within the IMGT/HLA database. Through subsequent multiple sequence alignments, a discernable set of unique sequences emerged from this process. Among these, a judicious selection was made of the eight most indicative sequences, which collectively constituted a targeted regional reference database. For a given sample, we extract reads

mapped to this region and methodically align them to this reference database. The two sequences commanding the highest rankings in terms of coverage and depth are then chosen for inclusion. These selected sequences are seamlessly integrated into the phased haplotypes. Within each haplotype, the sequence possessing the greatest aligned read count is identified. The net outcome of these procedures culminates in the comprehensive reconstruction of full-length diploid sequences of the *HLA-DRB1* locus.

Reporting HLA official designations

SpecHLA assigns each reconstructed sequence an official designation corresponding to the best-matched allele sourced from the IMGT/HLA database.[29] To achieve this assignment, a comprehensive comparison is conducted between the reconstructed sequence and all alleles documented in the database, leveraging Blastn. During this process, a sequence is annotated with the official designation of the allele exhibiting the highest identity to this sequence. In instances where multiple alleles share the same highest identity, the allele endowed with the highest ethnicity-dependent prior frequency is prioritized as the best-matched allele. Notably, all alleles achieving the highest identity are duly reported in the results. Furthermore, we undertake a direct alignment of the inferred HLA sequences with the exon database to extract G group resolution annotations.

SpecHLA provides five choices to determine how to use the population-dependent allele frequency, including *Asian*, *Black*, *Caucasian*, *Unknown*, and *nonuse*. We classify samples into *Asian*, *Black*, and *Caucasian* populations based on the geographical regions. The HLA allele population frequency table was downloaded from the Allele Frequency Net Database (http://www.allelefrequencies.net/). SpecHLA will use the population-specific allele frequency for designations if given *Asian*, *Black*, or *Caucasian*. SpecHLA will apply the mean frequency among the populations if given *Unknown* and will ignore the population frequency information if given *nonuse*. In the 183 WES samples from the 1000 Genomes project, we found that SpecHLA maintained consistent 2-field typing accuracy, irrespective of the inclusion or exclusion of population frequency information.

Detecting LOH events in cancer samples

To detect LOH events in cancer samples, we first compute haplotype frequencies (α_1 and α_2) with the genotype frequency of phased small variants. At each variant locus *j*, there are two alleles 0 and 1, and the frequency of allele 1 is denoted as β_j. β values can be observed from sequencing data and deduced from the haplotype. The observed allele frequency $\widehat{\beta_j}$ is obtained from the VCF file at each locus *j*. Moreover, we deduce the expected allele frequency β_j with phased genotype $g_j = (g_{j,1}, g_{j,2})$, where $g_{j,1}, g_{j,2} \in \{0, 1\}$, $g_{\cdot,1}$ are alleles on the first haplotype, and $g_{\cdot,2}$ are on the other haplotype. We have

$$\beta_j = g_{j,1} \times \alpha_1 + g_{j,2} \times \alpha_2. \quad \text{(Equation 9)}$$

The observed and expected β values should be the same in the ideal setting. To infer haplotype frequencies, we minimize the difference between observed and expected β values at all loci. Assume the number of small variant loci is *n*, and our optimization objective is

$$\text{argmin}_\alpha \sum_{j=1}^{n} \left(\beta_j - \widehat{\beta_j}\right)^2. \quad \text{(Equation 10)}$$

Based on the least squares method, the haplotype frequencies can be estimated by

$$\alpha_1 = \frac{\sum_{j=1}^{n}\left[g_{j,1} \times \widehat{\beta_j} + (1 - g_{j,1}) \times (1 - \widehat{\beta_j})\right]}{n}, \quad \text{(Equation 11)}$$

$$\alpha_2 = \frac{\sum_{j=1}^{n}\left[g_{j,2} \times \widehat{\beta_j} + (1 - g_{j,2}) \times (1 - \widehat{\beta_j})\right]}{n}. \quad \text{(Equation 12)}$$

Based on the inferred haplotype frequencies, we then calculate the copy number of two alleles (ε_1, ε_2) at each HLA locus. Calculating the copy number of HLA alleles should consider tumor purity because the cancer tissue is contaminated by normal cells. With tumor purity (ρ) and tumor ploidy (ψ) predicted by ABSOLUTE v1.2,[40] we have the following equations:

$$\varepsilon_1 + \varepsilon_2 = \psi, \quad \text{(Equation 13)}$$

$$\frac{\alpha_1}{\alpha_2} = \frac{\varepsilon_1 \times \rho + 1 - \rho}{\varepsilon_2 \times \rho + 1 - \rho}. \quad \text{(Equation 14)}$$

The copy number of the two alleles is then

$$\varepsilon_1 = \frac{\alpha_1 \times \rho \times \psi - \alpha_1 \times \rho + \alpha_2 \times \rho + \alpha_1 - \alpha_2}{\rho}, \quad \text{(Equation 15)}$$

$$\varepsilon_2 = \frac{\alpha_2 \times \rho \times \psi + \alpha_1 \times \rho - \alpha_2 \times \rho - \alpha_1 + \alpha_2}{\rho}. \quad \text{(Equation 16)}$$

The minor allele with a copy number less than 0.5 ($min(\varepsilon_1, \varepsilon_2) < 0.5$) is identified as the lost allele.

Long-read based HLA typing

Long reads are binned to the HLA loci using a method similar to short-read binning. Given the long-read data, SpecHLA aligns the reads to the HLA allele database using Minimap2[42] with the parameter -p 0.1 -N 100000 allowing multiple alignments. We then calculate the identity for each read alignment. If a read is mapped to the alleles of only a single HLA locus, we assign the read to the locus. If the read is mapped to several loci, we assign the read to a locus that (i) has the highest sequence identity and (ii) the identity difference between the locus and the second-highest-identity locus is greater than the value υ (υ defaults to 0.001).

We then map the binned reads to the IMGT representative reference of each locus using Minimap2[42] with the default parameter. Subsequently, we adopt Longshot v0.4.1[47] to detect and phase SNVs with the default setting. Since Longshot is unable to handle indels, the indels are ignored. We generate the diploid sequence of each gene using the consensus function of bcftools.[48] Finally, the diploid sequences are mapped to the IMGT/HLA database to obtain the official designations.

Benchmark datasets

1000 Genomes Project data

We downloaded 183 WES samples, 230 WGS samples, and 462 RNA-seq samples from the 1000 Genomes Project to evaluate SpecHLA. All the samples contain validated 2-field HLA typing results for *HLA-A, -B, -C,* and *-DRB1*; and 71 WGS, 127 WES, and 356 RNA-seq samples possess validated results for *HLA-DQB1*. The sequencing reads from these samples underwent refinement using the fastp tool v.0.20.0.[43] Furthermore, to validate SpecHLA's sequence reconstruction and pedigree relations integration abilities, we downloaded two family trios from the 1000 Genomes Project (NA12878-NA12891-NA121892 and NA19238-NA19239-NA19240).

IHWG cell line WES data

The IHWG cell line WES data were obtained from the NCBI BioProject with the accession number BioProject: PRJEB6763, and 89 of these samples have validated 2-field HLA typing results.[30] The mean sequencing depth of the MHC region is 59x in this dataset. To evaluate the robustness of SpecHLA, the data were subsampled at various sequencing depths, specifically at 10x, 15x, 20x, 30x, 35x, and 40x. This subsampling approach allows for the assessment of SpecHLA's performance under different sequencing depth conditions.

HGSVC2 project data

To assess SpecHLA in full-resolution HLA typing, we collected 32 WGS samples with PE data from the HGSVC2 project. Previous research has generated haplotype-resolved assemblies for these samples by combining the long-read technology and single-cell sequencing.[18] The haplotype-resolved assemblies were applied as ground truth. Further, seven out of the 32 samples possess matched PacBio HiFi data. We also collected the PacBio and ONT data for the sample NA12878. These samples were used to evaluate SpecHLA's ability in incorporating long reads.

To obtain the HLA allele sequences from the haplotype-resolved assemblies of HGSVC2 samples, we aligned each assembly onto the IMGT/HLA database using Minimap2.[42] As 20 samples have validated 2-field HLA types, we removed the alleles that did not fit the validated HLA types for these samples. Next, we identified the allele that best matched the assembly. Specifically, we collected the matched length and identity of each allele. If the allele with the longest matched length also had the highest identity, we selected it as the best-matched allele. However, if the allele with the longest matched length (A_m) differed from the allele with the highest identity (A_i), we used the following criteria to choose the best-matched allele: (i) If the identity score of A_m is less than 99.9%, we select A_i. (ii) If the matched length difference ratio of A_m and A_i is smaller than the identity difference ratio, we choose A_i. (iii) If the identity difference ratio between A_m and A_i is less than 0.5%, we select A_m. We only consider the subsequent criteria if the previous ones are not satisfied. Finally, we extracted the assembly sequence that mapped to the best-matched allele as the ground truth for sequence inference assessment.

Synthetic cancer data

We simulated cancer data by mixing exonic reads of cancer cell lines and matched normal cells to evaluate SpecHLA's LOH detection ability. We downloaded the WES data of three cancer cell lines (CRL-2314, CRL-5915, and CRL-5922) and matched normal cells.[27] We sub-sampled the cancer cell line data and the matched normal data and combined them to achieve different tumor purity (from 0.1 to 1, step by 0.1). Ten replicates were generated for each tumor purity. Together, it led to 300 samples; among them, there are 200 samples with LOH events, as only CRL-2314 and CRL-5922 have HLA LOH events.

Synthetic data of different proctocols

To validate SpecHLA, we simulated PE data with different sequencing parameters and generated 50 replicates for each parameter combination (sequencing depth: 10x, 20x, 30x, 50x, and 100x; read length: 75bp, 90bp, 100bp, and 150bp; sequencing error rate: 0% and 1%). For each sample, we included 31 homologous genes and pseudogenes such as *HLA-H* and *HLA-DRB3* (see Table S1). Two alleles were randomly selected from the IMGT/HLA database for each gene. Then DWGSIM was utilized to simulate PE

sequencing reads (https://github.com/nh13/DWGSIM). To assess SpecHLA in typing rare alleles, we simulated a dataset comprising HLA alleles that are not included in the lite IMGT/HLA database (50 replicates; sequencing depth: 100x; read length: 150bp; sequencing error rate: 0%). For the purpose of contrasting the performance of SpecHLA and HISAT-genotype in deducing new alleles, we simulated a set of 50 samples. These samples were created by introducing ten random SNVs to the IMGT representative reference, thus generating novel alleles for detection.

To evaluate the effectiveness of SpecHLA in incorporating genotype frequency, we conducted tests using three distinct datasets. Initially, we utilized the 200 LOH samples simulated from cancer cell lines, and subsequently simulated two additional datasets. In the first dataset, we simulated an allelic imbalance dataset with 10 replicates for each combination of parameters, including read lengths of 75bp, 90bp, 100bp, and 150bp, as well as sequencing error rates of 0% and 1%. This resulted in a total of 80 samples. Each gene within these samples consisted of one allele with a depth of 20x and another allele with a depth of 80x. The alleles were randomly selected from the database. For the second dataset, we introduced random SNVs to the IMGT reference allele at a rate of 0.2%, generating novel alleles. These novel alleles were then employed to simulate samples with varying allelic depths, specifically: *20x-80x*, *30x-70x*, *40x-60x*, *48x-52x*, and *50x-50x*. This simulation was performed 20 times, using a fixed read length of 75bp and a sequencing error rate of 0%. Consequently, a total of 100 samples were generated.

To test SpecHLA in adopting different sequencing protocols, we generated the sequencing reads of 10x, Hi-C, PacBio, and ONT. By introducing random SNVs at a rate of 0.1% to the IMGT representative reference, we effectively created new alleles, which in turn served as the basis for simulating a cohort of 50 individuals. Given the relatively sparse variant density, the challenge arose in acquiring ample phasing evidence for PE reads. Diverse read-generation protocols were then applied to each individual. We simulated the data of 10x, Hi-C, PacBio, and ONT using LRSIM,[41] sim3C,[46] PBSIM,[44] and NanoSim,[45] respectively (detailed parameters provided in Table S2). Minimap2[42] was applied for aligning PacBio and ONT reads. We used BWA MEM[35] with parameter -5SP to align Hi-C data and employed LongRanger (https://support.10xgenomics.com/genome-exome/software/downloads/latest) for aligning 10x linked reads.

Evaluation methods

Assessing 2-field resolution HLA typing accuracy

To guarantee an equitable comparison of HLA typing, we standardize the obtained typing results from different methodologies to the latest IMGT alleles, employing the most up-to-date file available (https://github.com/ANHIG/IMGTHLA/blob/Latest/Allelelist_history.txt). As the ground truth and inferred HLA types may contain ambiguities, we compare the reference HLA type tuple (R) with the inferred HLA type tuple (I) using a compatible function F, where $F(R,I)$ is 1 only if the intersection between R and I is not empty. For an HLA locus, suppose the reference HLA type tuples as R_1 and R_2, and the inferred HLA type tuples as I_1 and I_2. The number of correctly inferred alleles is calculated by

$$max\{F(R_1,I_1) + F(R_2,I_2), F(R_1,I_2) + F(R_2,I_1)\}. \quad \text{(Equation 17)}$$

The HLA type truth of the 1000 Genomes Project is at 2-field resolution; we thus compare the benchmark methods at 2-field resolution. All of the methods can provide HLA typing results with at least 2-field resolution, with the exception of Kourami, which only generates results at the G group resolution. To solve the problem, we collect all alleles corresponding to the G group resolution HLA type of Kourami to evaluate its accuracy at 2-field resolution.

Metrics for assessing reconstructed HLA sequences

To assess the reconstructed sequence, we align it to the true sequence using Blastn.[36] Since Blastn is a local alignment method, the alignment fragments might overlap. We remove the redundant alignment fragments. The number of mismatches (M) and the total number of gaps (G) on the non-redundant alignment fragments are counted. Assume the total length of the non-redundant alignment fragments as A. We measure the mismatches and gaps in the alignment using *mismatch rate* and *gap rate*, respectively, which are calculated by

$$mismatch\ rate = M/A, \quad \text{(Equation 18)}$$

$$gap\ rate = G/A. \quad \text{(Equation 19)}$$

Moreover, we define *sequence recall* and *sequence precision* to measure the inferred sequence's completeness and contamination. With the length of the true sequence (E) and the inferred sequence (L), sequence recall and sequence precision are deduced as

$$sequence\ recall = A/E, \quad \text{(Equation 20)}$$

$$sequence\ precision = A/L. \quad \text{(Equation 21)}$$

In addition, we employ edit distance to comprehensively measure the difference between the true and inferred sequences, which is obtained by the Python module edlib. We calculate the mean value of the diploid sequences in a sample for each metric.

Assessing HLA sequence reconstruction

We compared the full-resolution HLA typing utility of SpecHLA and HISAT-genotype in the HGSVC2 dataset. The extracted HLA allele sequences of HGSVC2 samples were used as ground truth to assess the sequence reconstruction accuracy. We counted the number of samples with reconstructed sequences for each HLA locus separately. In addition, we evaluated the exon reconstruction accuracy of SpecHLA in the HGSVC2 dataset. The alleles in the same G group have identical exons encoding the peptide binding domains (exon 2 and 3 for MHC class I and exon 2 only for MHC class II alleles). We compared the HLA typing accuracy of SpecHLA, Kourami, HISAT-genotype, HLA-VBSeq, HLA*LA, and HLA-HD at G group resolution. The methods were executed with default settings. The typing results of all methods were converted to G group resolution using the same G grouping file (date: 2023-01-12). The ground truth G group resolution HLA types were inferred by mapping the phased assemblies of HGSVC2 samples onto the HLA exons database using Minimap2.[42]

In assessing trio-consistency of two family trios, the exclusion of HISAT-genotype was necessary due to its inability to successfully recover alleles for all trio members of a given HLA locus, rendering an assessment of trio consistency unfeasible. To gauge sequence accuracy, we opted to select the most closely inferred sequence from the parental pool for each child's sequence. A comparative analysis of these two sequences was performed, employing metrics such as mismatch rate and gap rate. Notably, a lower mismatch rate and gap rate indicate a heightened level of trio consistency.

Additionally, the two trios were employed to assess SpecHLA's prowess in integrating pedigree relations for phasing. This evaluation involved separate runs of SpecHLA on the trios, both with and without pedigree relations. A subsequent comparison of sequence accuracy between these runs was undertaken. During the SpecHLA runs, the guidance of block linking from the database was intentionally omitted (-b 0). Moreover, to exemplify SpecHLA's ability to effectively integrate short and long reads, SpecHLA was also carried out with -b 0.

Computational resource comparison

We conducted a comparative analysis of the computational resource utilization across various HLA typing tools on a LINUX cluster featuring 8-core processors, a 143 MB cache, and a total of 16 TB global shared memory. We employed a random simulation involving a sample with PE150 reads, a sequencing depth of 50x, and a 1% sequencing error rate. Each tool was executed five times, and we documented both the CPU time and the peak RAM usage for each individual run of all the tools.

Benchmark tool parameter setting

We compared SpecHLA with ten state-of-the-art and widely used tools: Polysolver, OptiType, HLA-VBseq, Kourami, HISAT-genotype, HLA-HD, HLA*LA, arcasHLA, DASH, and LOHHLA. The default parameters were employed for running these tools (details provided in Table S3). Running LOHHLA to detect LOH was based on the HLA typing results of OptiType, as the authors of LOHHLA suggested. In the simulated datasets, the alleles were chosen from the database randomly, so we ran SpecHLA without considering the HLA population frequency using *nonuse*. We employed our read extraction procedure to obtain HLA-related reads for all the tools to save computational resources. The extracted reads were mapped to the HG19 reference as input for Polysolver and HLA*LA.

QUANTIFICATION AND STATISTICAL ANALYSIS

All procedures involving statistical analysis were described in the Method details section. The number of samples used in each experiment was described in both the figure legend and Results sections.

Cell Reports Methods, Volume *3*

Supplemental information

SpecHLA enables full-resolution HLA typing from sequencing data

Shuai Wang, Mengyao Wang, Lingxi Chen, Guangze Pan, Yanfei Wang, and Shuai Cheng Li

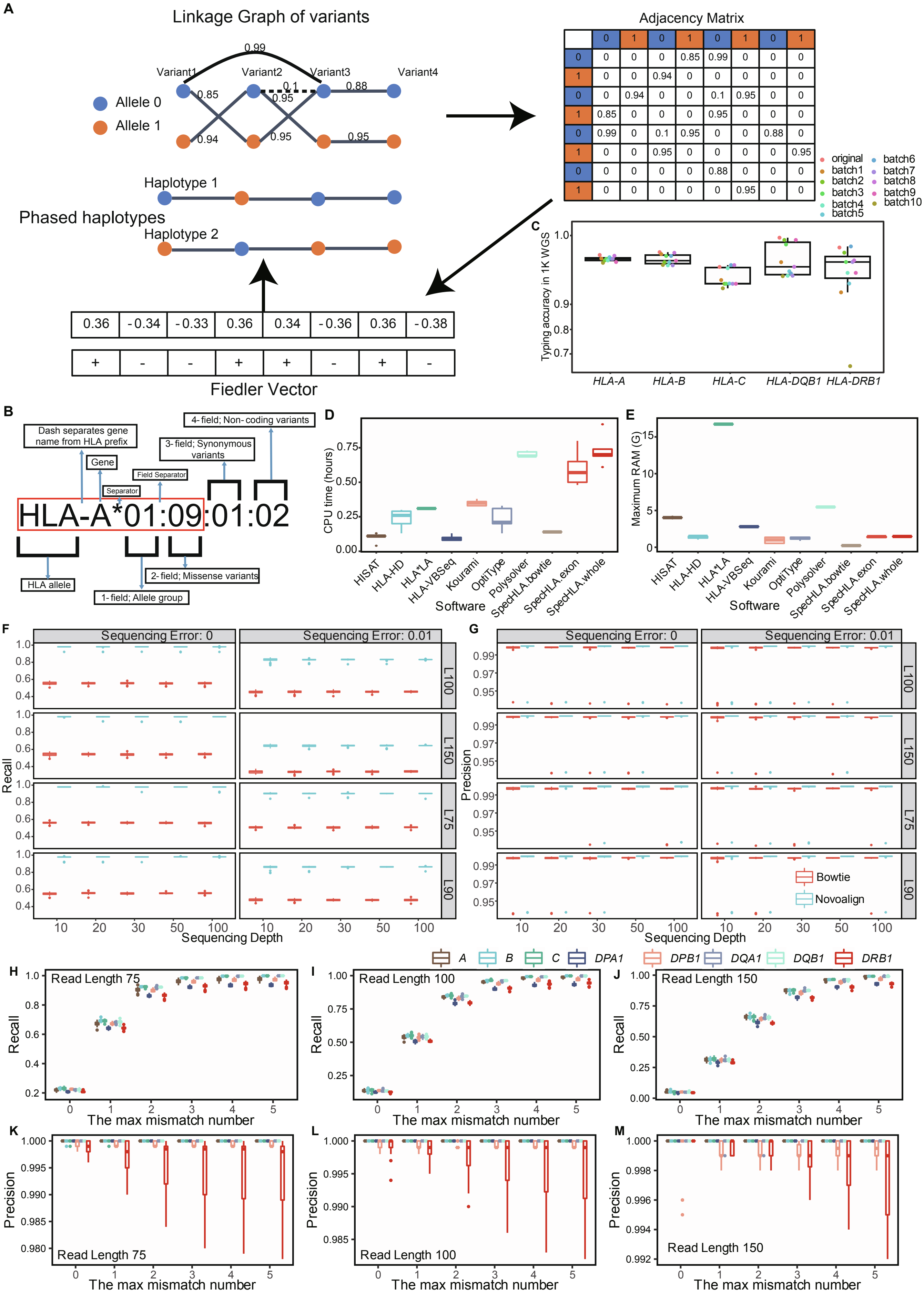

A
Linkage Graph of variants
Variant1
Variant2
Variant3
Variant4
Allele 0
Allele 1
Haplotype 1
Phased haplotypes
Haplotype 2
Adjacency Matrix
Fiedler Vector
0.36 -0.34 -0.33 0.36 0.34 -0.36 0.36 -0.38
+ - - + + - + -
B
HLA-A*01:09:01:02
Dash separates gene name from HLA prefix
Gene
Field Separator
Separator
3- field; Synonymous variants
4- field; Non- coding variants
HLA allele
1- field; Allele group
2- field; Missense variants
C
Typing accuracy in 1K WGS
HLA-A
HLA-B
HLA-C
HLA-DQB1
HLA-DRB1
original
batch1
batch2
batch3
batch4
batch5
batch6
batch7
batch8
batch9
batch10
D
CPU time (hours)
E
Maximum RAM (G)
Software
HISAT
HLA-HD
HLA*LA
HLA-VBSeq
Kourami
OptiType
Polysolver
SpecHLA.bowtie
SpecHLA.exon
SpecHLA.whole
F
G
Sequencing Error: 0
Sequencing Error: 0.01
Recall
Precision
L100
L150
L75
L90
Bowtie
Novoalign
Sequencing Depth
A
B
C
DPA1
DPB1
DQA1
DQB1
DRB1
H
I
J
Read Length 75
Read Length 100
Read Length 150
The max mismatch number
K
L
M

Figure S1: Illustration and evaluation of SpecHLA methodology, related to Figure 1 and STAR Methods.
(A) Illustration of variant phasing with the spectral graph theory. Alleles at each variant are represented as nodes (0 or 1), and the linkage between alleles is depicted as edges. The allele linkage between different variants can be inferred from the sequencing reads, database, genotype frequency, *etc.* The Fiedler vector of the adjacency matrix of the graph is calculated, and the haplotype is inferred by the sign of the Fiedler vector.
(B) Illustration of the naming scheme of HLA alleles. The 1-field indicates the allele group. The 2-field represents the specific HLA protein. The 3-field shows a synonymous DNA substitution within the coding region. The 4-field shows the variants in the non-coding region. Alleles that have identical exon sequences that encode the peptide binding domains will be assigned to the same G group. Full-resolution HLA type indicates the full DNA sequence of the HLA allele.
(C) Comparison of SpecHLA using different reference alleles in 230 WGS 1,000 Genomes samples. The y-axis represents the 2-field HLA typing accuracy, and the scales of 0.9-1.0 and 0.7-0.9 are different. The "*original*" group represents the IMGT representative reference, which concatenates the first recorded allele at each HLA locus. The "*batch**" groups refer to randomly selected alleles.
(D-E) Evaluation of the computational resource consumption of SpecHLA. Different HLA typing methods were run on a simulated sample five times, and the computational resource consumption of each run was measured by CPU time (D) and maximum RAM (E).
(F-G) Comparison of read-binning accuracy using Novoalign and Bowtie2 for alignment in 2,000 simulated samples. The read-binning accuracy was measured by recall (F) and precision (G).
(H-M) Evaluation of the read-binning performance with different maximum mismatch number thresholds (i.e., the hyper-parameter θ). In 200 simulated samples (sequencing depth: 100x; sequencing error rate 1%), we showed the recall with read lengths 75 (H), 100 (I), and 150 (J); and the precision with read lengths 75 (K), 100 (L), and 150 (M).

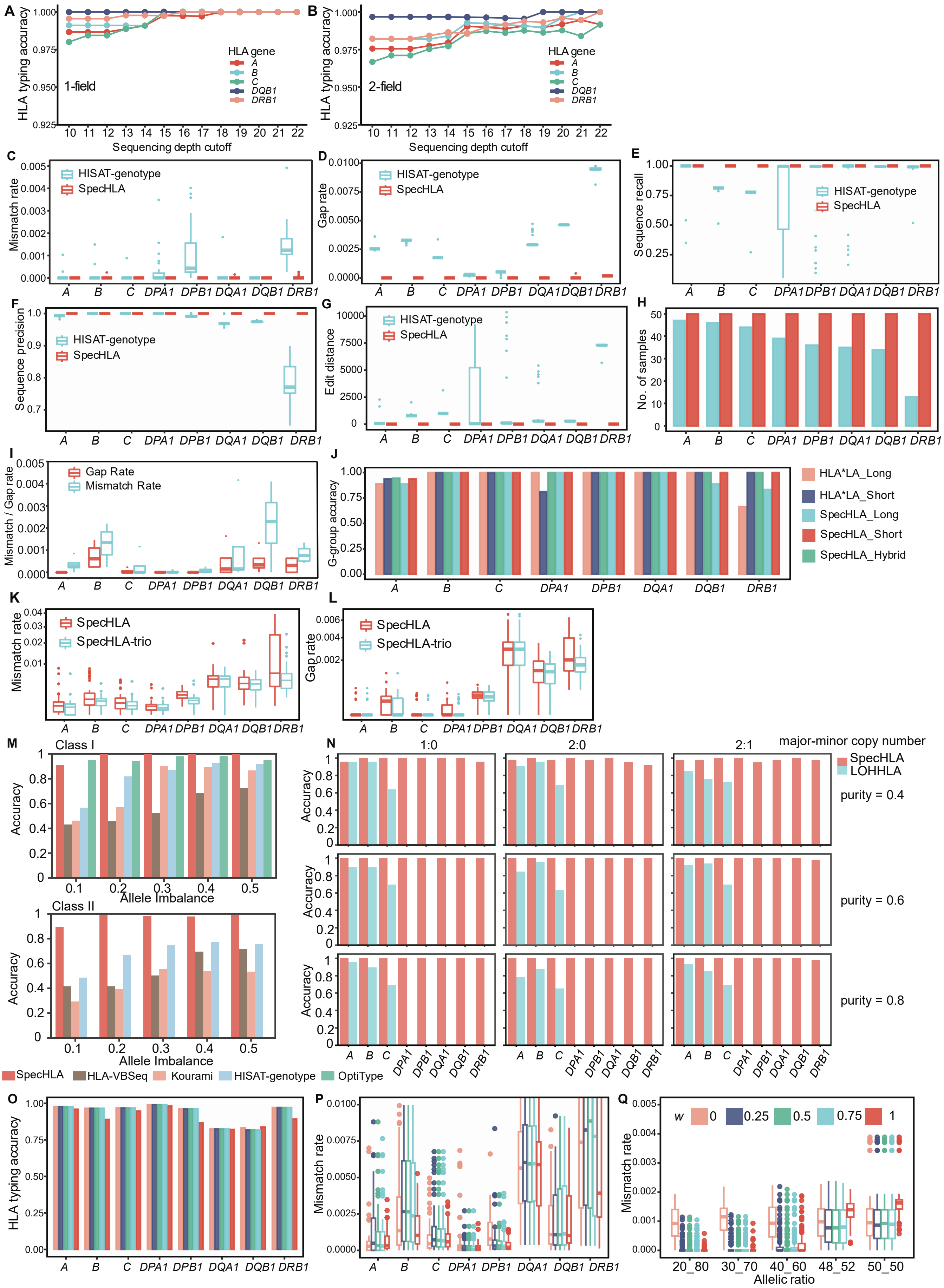

A
HLA typing accuracy
1-field
HLA gene
A
B
C
DQB1
DRB1
Sequencing depth cutoff
B
2-field
C
Mismatch rate
HISAT-genotype
SpecHLA
D
Gap rate
E
Sequence recall
F
Sequence precision
G
Edit distance
H
No. of samples
I
Gap Rate
Mismatch Rate
Mismatch / Gap rate
J
G-group accuracy
HLA*LA_Long
HLA*LA_Short
SpecHLA_Long
SpecHLA_Short
SpecHLA_Hybrid
K
SpecHLA
SpecHLA-trio
L
M
Class I
Accuracy
Allele Imbalance
Class II
SpecHLA
HLA-VBSeq
Kourami
HISAT-genotype
OptiType
N
1:0
2:0
2:1
major-minor copy number
SpecHLA
LOHHLA
purity = 0.4
purity = 0.6
purity = 0.8
O
P
Q
w
Allelic ratio

Figure S2: Additional evaluation of SpecHLA for HLA typing and LOH inference, related to Figure 2-4 and STAR Methods.
(A-B) Evaluation of SpecHLA using different sequencing depth cutoffs in 230 WGS samples of the 1,000 Genomes Project. The 1-field (A) and 2-field (B) HLA typing accuracy of different genes were displayed separately. Only samples with a sequencing depth higher than the cutoff were included in the evaluation.
(C-H) Comparison of SpecHLA and HISAT-genotype for inferring novel allele sequences in 50 simulated samples. The reconstructed novel alleles were measured by mismatch rate (C), gap rate (D), sequence recall (E), sequence precision (F), and edit distance (G). The number of samples with reconstructed diploid sequences for each gene is shown in (H).
(I) Assessment of trio consistency of SpecHLA in two family trios of the 1,000 Genomes Project. The difference between the inferred sequences of children and parents was measured by mismatch rate and gap rate.
(J) G-group resolution typing accuracy of HLA*LA and SpecHLA using different data protocols in seven HGSVC2 samples and NA12878. HLA*LA was performed on short reads ("*HLA*LA_Short*") and long reads ("*HLA*LA_Long*") separately, while SpecHLA was run with short reads ("*SpecHLA_Short*"), long reads ("*SpecHLA_Long*"), and both short and long reads ("*SpecHLA_Hybrid*").
(K-L) Validation of SpecHLA for incorporating pedigree information using 50 simulated family trios. The performance was measured by mismatch rate (K) and gap rate (L). The y-axis is scaled by the square root. "*SpecHLA-trio*" indicates the incorporation of pedigree information and "*SpecHLA*" means only using reads.
(M) Evaluation of SpecHLA's robustness in allelic imbalance samples using 250 simulated samples. The data had various minor allele ratios (0.1, 0.2, 0.3, 0.4, and 0.5), with 50 replicates for each ratio. The 2-field HLA typing accuracy of MHC class I and II genes was shown separately.
(N) Evaluation of the LOH detection of SpecHLA with different tumor purity and ploidy using simulated data. The LOH detection accuracy of LOHHLA and SpecHLA was compared in a total of 450 samples from nine PE datasets with different tumor purity (0.4, 0.6, 0.8) and major-minor copy numbers (1:0, 2:0, 2:1), with 50 replicates for each dataset (sequencing depth: 100x; read length: 150bp; sequencing error rate: 0%).
(O-Q) Evaluation of SpecHLA with different values of the hyper-parameter *w*. The hyper-parameter *w* adjusts the weight of genotype frequency information. (O) 2-field HLA typing accuracy in 200 LOH samples generated by cancer cell lines and normal cell data. (P) Mismatch rate in 80 allelic imbalance simulated samples. In each sample, the depths of the two haplotypes were 20x and 80x. (Q) Mismatch rate of SpecHLA in 100 simulated novel-allele samples with different allelic depths. E.g., "*30_70*" shows the depths of the two haplotypes were 30x and 70x.

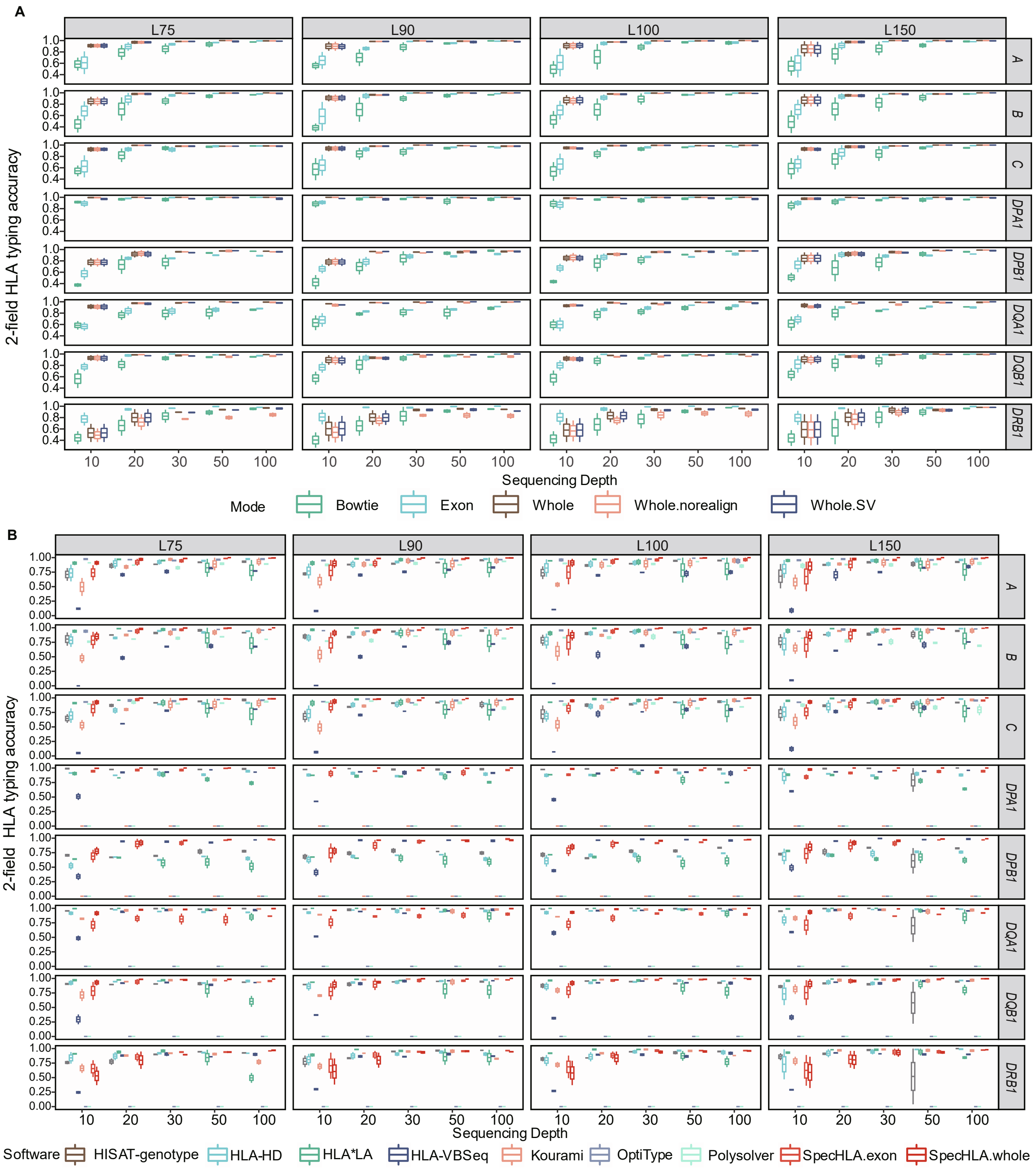

Figure S3: Evaluation of SpecHLA for 2-field typing using the 2,000 simulated samples, related to Figure 2. **(A)** Comparison of SpecHLA with different modes for 2-field typing. The 2-field typing accuracy of SpecHLA in different modes with varying sequencing depths and read lengths was displayed separately. "*Bowtie*" indicates the usage of Bowtie2 for alignment in the read binning step. "*Exon*" and "*whole*" represent focusing on the exon region and the full-length allele, respectively. "*Whole.norealign*" denotes discarding the local assembly step, while "*Whole.SV*" involves considering long InDels.

(B) Comparison of SpecHLA and other methods for 2-field typing. The 2-field typing accuracy of different software with different sequencing depths and read lengths was displayed separately. "*SpecHLA.exon*" refers to reconstructing exons and obtaining typing annotation based on exons, while "*SpecHLA.whole*" focuses on the full-length HLA alleles.

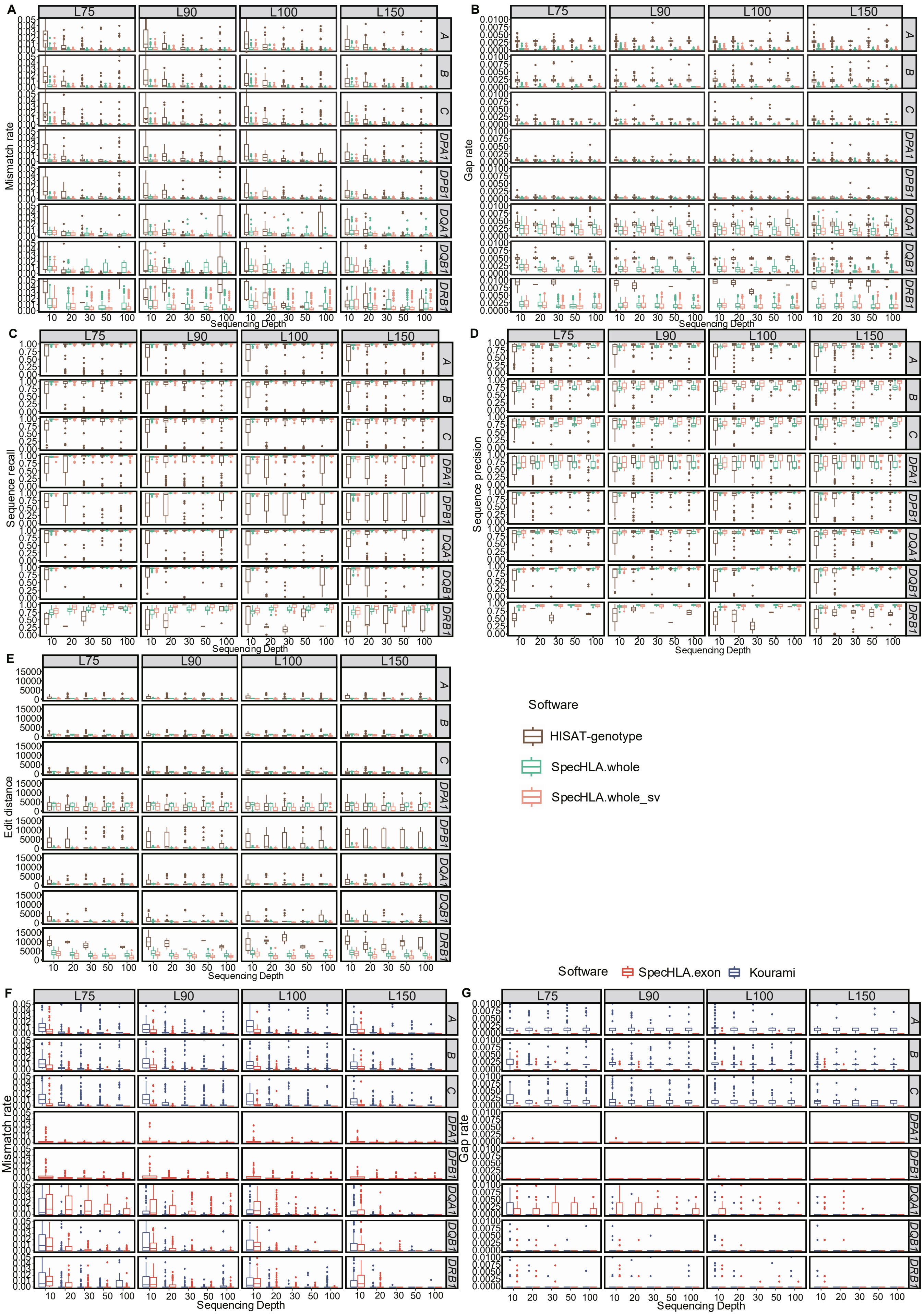
A
B
C
D
E
F
G
L75
L90
L100
L150
A
B
C
DPA1
DPB1
DQA1
DQB1
DRB1
Mismatch rate
Gap rate
Sequence recall
Sequence precision
Edit distance
Sequencing Depth
10
20
30
50
100
Software
HISAT-genotype
SpecHLA.whole
SpecHLA.whole_sv
SpecHLA.exon
Kourami

Figure S4: Evaluation of SpecHLA for sequence reconstruction using the 2,000 simulated samples, related to Figure 2.
(A-E) Comparison of SpecHLA and HISAT-genotype for full-resolution HLA typing. The performance was measured by mismatch rate (A), gap rate (B), sequence recall (C), sequence precision (D), and edit distance (E). The accuracy was calculated for different read lengths and sequencing depths separately. "*SpecHLA.whole*" denotes performing HLA typing on full-length allele while ignoring long InDels, and "*SpecHLA.whole_sv*" involves identifying and phasing long InDels.
(F-G) Comparison of SpecHLA (exon mode) and Kourami for exonic sequence inference. The exonic sequence was assessed by mismatch rate (F) and gap rate (G). The accuracy was calculated for different read lengths and sequencing depths separately. Only exons 2 and 3 for MHC class I genes and exon 2 for MHC class II genes were considered.

Gene	No. of alleles (exon)	No. of alleles (full-length)	Representative Allele	Highly divergent regions
HLA-A	131	1141	A*01:01:01:01	
HLA-B	230	1512	B*07:02:01:01	1500-1800
HLA-C	80	1323	C*01:02:01:01	
HLA-DMA	2	5		
HLA-DMB	2	8		
HLA-DOA	1	7		
HLA-DOB	3	10		
HLA-DPA1	4	86	DPA1*01:03:01:01	
HLA-DPA2	2	5		
HLA-DPB1	55	441	DPB1*01:01:01:01	2150-2800, 9724-9950, 10000-10500
HLA-DPB2	2	5		
HLA-DQA1	16	140	DQA1*01:01:01:01	2900-3202, 3345-3812, 3900-4150, 4999-5320, 5800-6150, 6550-6800
HLA-DQA2	7	15		
HLA-DQB1	24	239	DQB1*02:01:01	2450-2650, 3205-4115, 4100-4250, 4400-4600, 4750-5200, 5300-5700, 5800-6100
HLA-DRA	1	28		
HLA-DRB1	816	816	DRB1*01:02:01:01	2250-2450, 2774-3266, 3292-5120, 6700-7100, 7192-7540, 8893-9299, 10032-10305, 11020-11728
HLA-DRB3	77	77		
HLA-DRB4	24	24		
HLA-DRB5	26	26		
HLA-E	10	41		
HLA-F	6	44		
HLA-G	6	40		
HLA-H	6	9		
HLA-HFE	1	3		
HLA-J	2	9		
HLA-K	3	6		
HLA-L	2	5		
HLA-MICA	13	15		
HLA-MICB	12	31		
HLA-N	1	5		
HLA-P	3	5		
HLA-S	4	7		
HLA-T	3	8		
HLA-TAP1	2	6		
HLA-TAP2	1	8		
HLA-U	4	5		
HLA-V	1	3		
HLA-W	5	11		
HLA-Y	3	3		

Table S1: HLA database profile used for the reads binning and reads alignment procedures of SpecHLA, related to STAR Methods.
The first column presents the 39 HLA genes included in the HLA database. The second and third columns display the numbers of alleles in the HLA database for reads binning of exon typing and full-length typing, respectively. After downloading the IMGT/HLA database, alleles with a population frequency equal to zero were removed for the eight focused genes, while all alleles were retained for other genes. Additionally, alleles of *HLA-DRB2, -DRB6, -DRB7, -DRB8,* and *-DRB9* were discarded. For exon typing, the database retains only one allele from the alleles with the same 2-field designation for each HLA gene, except for *HLA-DRB1*. The fourth column indicates the IMGT representative alleles used in the read-binning process. The assigned reads are separately projected to the representative allele of each gene. The last column shows the interval of highly divergent regions of the IMGT representative alleles. SpecHLA assembles the reads mapped to such regions to enhance the quality of read alignment.

Data Protocol	Software	Command
PE	DWGSIM v0.1.13	dwgsim -e 0 -E 0 -1 150 -2 150 -C 10 -r 0
10x	LRSIM v1.0	perl simulateLinkedReads.pl -r -p -x 1 -f 3 -t 1 -m 10 -o -c LRSIM/test/fragmentSizesList
Hi-C	sim3C v0.2	sim3C –dist uniform -n 37574 -l 150 -e NlaIII -m hic
PacBio	PBSIM v1.0.3	pbsim –data-type CLR –seed 88 –accuracy-mean 0.85 –accuracy-min 0.80 –depth 10 –model_qc model_qc_clr
ONT	NanoSim v3.1.0	simulator.py genome -c human_NA12878_DNA_FAB49712_guppy/training -rg -o -max 5000 -n 4000 –seed 66

Table S2: Parameters for simulating different data protocols, related to STAR Methods. We simulated data using these protocols for each one of the 50 simulated individuals.

Software	Version	Data	Parameter
SpecHLA	v1.0.1	WGS	-u 0 -p $pop -m 5
		WES	-u 1 -p $pop -k 4
		RNA-seq	-u 1 -p $pop
		HGSVC2 Full-length	-u 0 -p nonuse
		HGSVC2 hybrid	-u 0 -b 0 -1 $fq1 -2 $fq2 -t $pacbio
		trios for trio-consistency assessment	-u 0 -m 4
		trios for pedigree info integration	-u 0 -m 4 -b 0
		Simulated data	-u 0 -q 1 -s 3 -r 0.1 -p nonuse
		Simulated data using rare alleles	-u 0 -p nonuse -m 10
		long-read only	long_read_typing.py -r $long_reads -n $sample
		IHWG cell line	-u 1 -s 3 -m 5 -k 3 -z True
		LOH	-u 1 -r 0.05 -w 0.1 -p Unknown
HISAT-genotype	v1.3.2	Simulated data/HGSVC2	–base hla –locus-list A,B,C,DPA1,DPB1,DQA1,DQB1,DRB1 –assembly -1 $fq1 -2 $fq2 –read-len $readlen
		WGS/WES/RNA-seq	–base hla –locus-list A,B,C,DPA1,DPB1,DQA1,DQB1,DRB1 -1 $fq1 -2 $fq2 –read-len $readlen
HLA-HD	v1.4.0	WGS/WES/RNA-seq/Simulated data	-m $readlen -c 0.95
HLA-LA	v1.0.2	WGS/WES/Simulated data	default
		long-read	–longReads pacbio
HLA-VBseq	v2	WGS/WES/Simulated data	–alpha_zero 0.01 –is_paired -d 4
Kourami	v0.9.6	WGS/WES/simualtion	default
OptiType	v1.3.1	WGS/WES/Simulated data	default
		RNA-seq	–rna
Polysolver	v1.0.0	WES/Simulated data	default
arcasHLA	v0.5.0	RNA-seq	arcasHLA genotype $fq1 $fq2 -g A,B,C,DPA1,DPB1,DQB1,DQA1,DRB1
LOHHLA	v1.1.6	LOH	default
DASH	training.xgboost_model. 2021_05_10.p	LOH	default

Table S3: Parameter settings of benchmark tools for HLA typing and LOH detection, related to STAR Methods.
For all benchmark tools, unless mentioned otherwise, any unmentioned parameters were set to their default values.
